# Inhibition of the Smc5/6 Complex during Meiosis Perturbs Joint Molecule Formation and Resolution without Significantly Changing Crossover or Non-crossover Levels

**DOI:** 10.1371/journal.pgen.1003898

**Published:** 2013-11-07

**Authors:** Ingrid Lilienthal, Takaharu Kanno, Camilla Sjögren

**Affiliations:** Karolinska Institutet, Department of Cell and Molecular Biology, Stockholm, Sweden; State University of New York, United States of America

## Abstract

Meiosis is a specialized cell division used by diploid organisms to form haploid gametes for sexual reproduction. Central to this reductive division is repair of endogenous DNA double-strand breaks (DSBs) induced by the meiosis-specific enzyme Spo11. These DSBs are repaired in a process called homologous recombination using the sister chromatid or the homologous chromosome as a repair template, with the homolog being the preferred substrate during meiosis. Specific products of inter-homolog recombination, called crossovers, are essential for proper homolog segregation at the first meiotic nuclear division in budding yeast and mice. This study identifies an essential role for the conserved Structural Maintenance of Chromosomes (SMC) 5/6 protein complex during meiotic recombination in budding yeast. Meiosis-specific *smc5/6* mutants experience a block in DNA segregation without hindering meiotic progression. Establishment and removal of meiotic sister chromatid cohesin are independent of functional Smc6 protein. *smc6* mutants also have normal levels of DSB formation and repair. Eliminating DSBs rescues the segregation block in *smc5/6* mutants, suggesting that the complex has a function during meiotic recombination. Accordingly, *smc6* mutants accumulate high levels of recombination intermediates in the form of joint molecules. Many of these joint molecules are formed between sister chromatids, which is not normally observed in wild-type cells. The normal formation of crossovers in *smc6* mutants supports the notion that mainly inter-sister joint molecule resolution is impaired. In addition, return-to-function studies indicate that the Smc5/6 complex performs its most important functions during joint molecule resolution without influencing crossover formation. These results suggest that the Smc5/6 complex aids primarily in the resolution of joint molecules formed outside of canonical inter-homolog pathways.

## Author Summary

Most eukaryotic cells are diploid, which means that they contain two copies of each chromosome – one from each parent. In order to preserve the chromosome number from generation to generation, diploid organisms employ a process called meiosis to form gametes containing only one copy of each chromosome. During sexual reproduction, two gametes (sperm and eggs in mammals) fuse to form a zygote with the same chromosome number as the parents. This zygote will develop into a new organism that has genetic characteristics unique from, but still related to, both parents. The reduction of chromosome number and the reshuffling of genetic traits during meiosis depend on the repair of naturally occurring DNA breaks. Improper break repair during meiosis may block meiosis altogether or form genetically instable gametes, leading to fertility problems or defects in the offspring. The study presented here demonstrates the importance of the evolutionarily conserved Smc5/6 protein complex in upholding the integrity of meiotic repair processes. Our results show that cells deficient in components of the Smc5/6 complex lead to inviable meiotic products. Cells lacking functional Smc5/6 complex are unable to direct DNA repair to the proper template and accumulate abnormal repair intermediates, which inhibit the reductive division.

## Introduction

Meiosis is the cell division by which haploid gametes are created in sexually reproducing organisms. It is specialized to preserve the chromosome number among generations and to create genetic diversity in a population. Meiosis begins with the replication of each homologous parental chromosome (homolog) into a pair of sister chromatids. Two sequential rounds of DNA segregation then follow. The first, MI, segregates the homologs away from each other, while the second, MII, separates the sister chromatids. This leads to the formation of four haploid cells from a single diploid parent. Prior to homolog segregation, programmed DNA double-strand breaks (DSBs) are induced that are repaired through a process called homologous recombination. In budding yeast and mice, recombination is essential for proper homolog segregation at MI. Together with sister chromatid cohesion, recombination facilitates segregation by creating stable attachments between the maternal and paternal homologs, thus ensuring their correct organization in preparation for anaphase I [1].

Meiotic DSBs are catalyzed by the enzyme Spo11 [2], [3]. After DSB induction, the ends of the DSB are resected to form single-stranded DNA overhangs that can invade a homologous sequence for repair. An initial DNA joint molecule (JM) is then formed following exchange of the broken end with a homologous sequence (Figure S1). The JM is further processed and enzymatically resolved according to its composition to generate two types of products: Those that mutually exchange DNA sequences between the homologs to physically attach them, called crossovers (COs), and those that repair without mutual exchange, called non-crossovers (NCOs) [4], [5]. Initial stabilization after invasion of the break end forms a transient JM called a single-end invasion (SEI) (Figure S1) [6]. Displacement of the invading strand of the SEI, such as in helicase-mediated unwinding by the BLM ortholog Sgs1, followed by ligation with the free DSB end, forms a NCO in a process called synthesis-dependent strand annealing (SDSA) (Figure S1A) [6]–[8]. Alternatively, the SEI can be stabilized and processed to form a stable JM intermediate known as a double-Holliday junction (dHJ) (Figure S1B) [9]. The dHJ must be cleaved by endonucleases or dissolved using a helicase in combination with a topoisomerase in order to be processed into its products [5], [10]. During meiosis, NCOs and dHJ-JMs form concurrently while COs form after dHJ disappearance, indicating that COs are the main products of dHJ resolution (Figure S1C) [11], [12]. NCOs, on the other hand, are primarily formed via SDSA [11]. Regulating the formation and resolution of dHJ-JMs is essential for homolog segregation at MI, and several factors have been identified that specifically promote CO formation without influencing overall DSB repair [10]. Most of these proteins belong to the meiosis-specific ZMM (Zip1-4, Mer3, Msh4, Msh5, Spo16) family, which stimulate COs by stabilizing dHJ formation [13]–[15]. The phosphatase PP4 (Pph3/Psy2) also promotes proper CO formation by stabilizing SEIs [16]. Moreover, recent evidence has implicated the mismatch repair components Exo1 and the MutLγ complex Mlh1–Mlh3 as crossover-specific JM resolution factors [17]. While the ZMM proteins regulate the majority of COs in budding yeast and mice, a subset is dependent on the endonuclease Mus81-Mms4 [18]–[20]. In fission yeast, however, all COs form via the Mus81-Eme1 (Mus81-Mms4 in budding yeast) pathway and are derived from single, rather than double, HJs [21]–[24].

Chromatin in budding yeast is organized in a loop-axis configuration [25]. Meiotic DSB hotspots are located in the DNA loops while recombination is carried out close to the meiotic axis [26], [27]. Normal DSB induction is dependent of the tethering of DSB hotspot sequences to accessory DSB proteins at the axis prior to break induction [28]–[30]. Hence, proper meiotic recombination relies on correct loop-axis configuration and events that change this architecture alter recombination events and outcomes [27]–[31]. Despite the presence of the sister chromatid, the homologous chromosome is preferred as a repair template during meiosis [32]. This inter-homolog (IH) bias is due to combined efforts of mechanisms that promote invasion of the homolog strand, and components of the meiotic axis that physically block sister invasion [32]–[37]. The meiotic axis includes the cohesin subunit Rec8, which is required for proper axis formation and loop organization [38]. In the absence of Rec8, the loop-axis configuration is perturbed and DSBs form at low levels with altered distribution as compared to wild-type cells [39],[40]. The axis-organizing function of Rec8 is also needed to maintain IH bias during the SEI-to-dHJ transition, even though Rec8 is actually a promoter of inter-sister (IS) recombination most likely due to its role in sister chromatid cohesion (see below) [40]. The presence of IH-promoting axis components antagonize the IS bias created by Rec8 to allow IH events to dominate [40]. To further promote IH-recombination, the ZMM proteins form a structure called the synaptonemal complex (SC) between the homologs. The SC holds the homologs close to one another during recombination, thereby facilitating homolog-directed strand invasion [14],[15]. Together these mechanisms establish a bias for IH recombination but do not eliminate IS recombination, with the possibility that up to one-third of all wild-type recombination events may be directed to the sister [40],[41]. These IS repair events rarely go via a detectable JM intermediate, most likely due to the decreased preference for recombination via a IS-JM in combination with fast turnover rates for IS-JMs that may arise [42]. If inefficiently resolved, an inter-sister DNA link on the telomere-proximal side of a CO will inhibit the segregation of homologs at MI, making it crucial for cells to properly process inter-sister recombination events.

As stated, the cohesin complex is a meiotic axis component required for proper recombination. It is also essential for sister chromatid cohesion during mitosis and meiosis [43]. Cohesin is a member of the evolutionarily conserved structural maintenance of chromosomes (SMC) family of proteins, which also includes the Smc5/6 complex. Components of the Smc5/6 complex were first identified as repair proteins working in the homologous recombination pathway [44]–[46]. The complex consists of eight subunits: Smc5, Smc6, Nse1, Mms21 (Nse2) and Nse3-6, and assists in the reduction of topological stress during replication as well as DSB repair in post-replicative vegetative cells [46]–[50]. Cells harboring mutations in *SMC5*, *SMC6* or *MMS21* accumulate recombination intermediates following DNA damage inflicted during mitotic S phase [51]–[53]. Mutating genes involved in the resolution of aberrant recombination structures at blocked replication forks, such as *MUS81-MM4*, *SGS1* and *TOP3*, aggravates this phenotype [54]–[56]. Recent studies have pointed to a role for the Smc5/6 complex during meiotic recombination as well. A study in *C. elegans* showed that the Smc5 and Smc6 proteins are required to process recombination structures in germ line cells [57]. In fission yeast, *nse1-3* are needed for proper meiotic chromosome segregation [58] [59]. In addition, fission yeast cells harboring mutations in *nse6* accumulate meiotic JMs in the form of single HJs that resemble those found in cells lacking the endonuclease Mus81 [59]. Although the HJs are DSB-dependent, the *nse6* mutant used in this study was not meiosis-specific and accumulated recombination intermediates during mitosis and pre-meiotic S phase as well [59]. Thus, the meiotic intermediates observed may have been a consequence of lesions accumulated prior to meiotic induction. A study in budding yeast was also unable to isolate a meiosis-specific phenotype for mutants of the Smc5/6 protein complex. The segregation block in these *smc6* mutants was *not* DSB-dependent and most likely caused by defects accumulated during mitosis or pre-meiotic S-phase [60]. Due to the discrepancies between these studies, the meiotic function of the Smc5/6 protein complex remains unclear.

In this study, we employed meiosis-specific alleles of genes encoding for the Smc5/6 complex to investigate the meiotic role of the complex in the budding yeast *Saccharomyces cerevisiae*. Cells lacking components of the Smc5/6 complex during meiosis experience a segregation block that is dependent on DSB formation. Mutants are normal in meiotic prophase progression and DSB repair and have no significant defects in sister chromatid cohesion. Return-to-function studies indicate that the complex works at later stages of meiotic recombination. This function is most critical at times of JM resolution, and cells with non-functional Smc6 accumulate high levels of JMs in the form of both IS- and IH-JM intermediates. CO and NCO levels remain unchanged, indicating that the majority of IH-JMs are processed normally, and suggesting that most of the unresolved JM intermediates are derived from inter-sister recombination events. These findings demonstrate that the Smc5/6 protein complex is directly involved in meiotic recombination and suggest that Smc6 plays a key role in resolving recombination intermediates during meiosis, especially those that form between sister chromatids.

## Results

### The Smc5/6 protein complex is required for meiotic chromosome segregation

**Figure 1:**
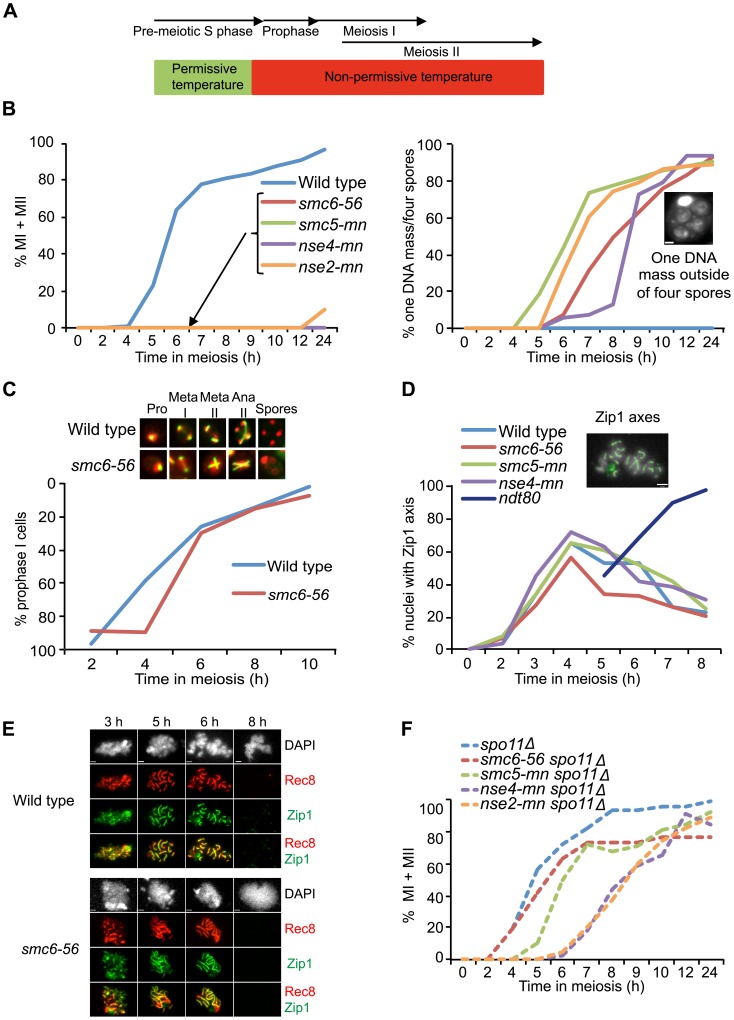
Smc5/6 mutations cause a recombination-dependent segregation block without affecting meiotic progression. (**A**) Set-up of the soft-shift method. Cells were shifted to non-permissive temperature (33°C) upon completion of pre-meiotic S phase as judged from FACS profiles shown in Figure S2. (**B**) Meiotic time courses for wild-type (CB1017), *smc6-56* (CB1032), *smc5-mn* (CB1872), *nse4-mn* (CB1511) and *nse2-mn* (CB2053) strains. At indicated times, cells were fixed and stained with DAPI to determine their nuclear content. Percent of MI+MII cells shown at left, percent of cells with one DNA mass outside four empty spores shown at right. Inset picture illustrates the “one DNA mass outside of four empty spores” phenotype, scale bar = 1 µm. Graphs represent a single synchronous meiotic time course. N = 200. (**C**) Meiotic spindle formation in wild type (CB1017) and *smc6-56* (CB1032). Fixed whole cells were stained with an anti-α-tubulin antibody (green) and DAPI (red). The images represent spindle morphology in wild type and *smc6-56* at prophase (pro), metaphase I (meta I), metaphase II (meta II), anaphase II (ana II) and after completing sporulation (spores). Meiotic progression demonstrated by plotting the fraction of cells with a single tubulin focus remaining at each time point on an inverted y-axis. N = 200. (**D**) Meiotic progression determined as percent of nuclei with full or partial Zip1 axes analyzed on meiotic spreads at indicated times. Picture demonstrates full Zip1 axes shown in green, scale bar = 2 µm. Dark blue line shows Zip1 axis formation when in the absence of *NDT80* function. N = 100. (**E**) *smc6-56* (CB1346) and wild-type (CB46) cells undergoing meiosis under soft-shift conditions were isolated and surface-spread to detect Zip1 (green) and epitope-tagged Rec8 (red). DNA was visualized with DAPI (gray). Scale bar = 1.8 µm. (**F**) Meiotic progression in *spo11Δ* (CB1302), *spo11Δ smc6-56* (CB1301), *spo11Δ sm5-mn* (CB1754), *spo11Δ nse4-mn* (CB1510) and *spo11Δ nse2-mn* (CB2067) shown in percent of MI+MII cells. N = 200.

To initially address the meiosis-specific function of the Smc5/6 protein complex, the temperature-sensitive *smc6-56* allele was utilized. This mutant has known mitotic recombination defects at high temperature [51],[61]. At permissive temperature, *smc6-56* cells underwent normal meiotic divisions and formed viable spores (Figure S2A). When meiosis was carried out at non-permissive temperature from the time of meiotic induction, the *smc6-56* mutant exhibited a mixture of two phenotypes: cells that did not appear to have entered the meiotic program and accumulated as mononucleates, and cells that failed to segregate chromosomes but formed spores (Figure S2B). A mixed cell population was also observed in a previous study when cells with the temperature-sensitive allele *smc6-9* were grown at non-permissive temperature from the time of meiotic induction [60]. The authors of this paper concluded that the meiotic defects in *smc6-9* cells were largely due to problems acquired during mitosis or pre-meiotic S phase [60]. The mononucleate population in the *smc6-9* and *smc6-56* mutants resembles that observed in mitotic cells harboring the *smc6-56* allele, in which approximately half of the cells arrest in G2/M after replication at non-permissive temperature [49]. To focus on non-replicative meiotic functions, *smc6-56* cells were allowed to complete pre-meiotic replication at permissive temperature before shifting to non-permissive temperature (Figure 1A, Figure S2C). Under such “soft-shift” conditions, the *smc6-56* mutant only formed cells containing one unsegregated DNA mass outside four empty spores (Figure 1B). To confirm that this defect was not caused by high temperature and reflected the true meiotic phenotype of a *smc5/6* mutant, meiotic-null (mn) alleles of *SMC5*, *NSE4* and *NSE2* were constructed by replacing their endogenous promoters with the mitosis-specific *CLB2* promoter [62]. Because *CLB2* is not down-regulated until after pre-meiotic S phase, replication defects were avoided using this system [63],[64]. As in the *smc6-56* mutant, *smc5-mn*, *nse4-mn* and *nse2-mn* mutants were not able to segregate their chromosomes and instead formed cells with one DNA mass outside of four empty spores (Figure 1B). This demonstrates that the *smc6-56* phenotype reflects a meiotic function of the Smc5/6 complex.

### Mutants of the Smc5/6 complex complete the meiotic program

In *S. cerevisiae*, spores form around duplicated spindle pole bodies regardless of DNA location [65]. Thus the “one DNA mass outside of four empty spores” phenotype of *smc5/6* mutants suggests that they complete the meiotic program. To test this hypothesis, spindle morphology was monitored in the *smc6-56* mutant. In line with the idea that *smc5/6* mutants do not hinder meiotic progression, *smc6-56* cells were able to duplicate their spindle-pole bodies and elongate their spindles despite abnormal spindle morphology due to failure to segregate the DNA (Figure 1C). To further challenge the assumption that *smc5/6* cells complete the meiotic program, meiotic progression was analyzed by scoring the dynamics of Zip1 axes. Zip1 is a ZMM component of the SC [66]. Cells that are unable to complete recombination form incomplete Zip1 axes and do not progress past prophase [67]. Mutants lacking the transcription factor Ndt80 can initiate recombination but fail to signal downstream factors necessary to complete recombination and exit prophase and accumulate with full Zip1 axes [68]–[70]. Zip1 axes were formed and removed normally in *smc6-56*, *smc5-mn* and *nse4-mn* mutants (Figure 1D). The *smc6-56* mutant was also normal in the timing and morphology of Zip1 and Rec8 axes (Figure 1E). Together, these data demonstrate that cells lacking Smc5/6 components fail to segregate their DNA but do not halt the meiotic cell cycle.

### The segregation block in Smc5/6 mutants is dependent on meiotic recombination

To test if the segregation block in Smc5/6 complex mutants was due to meiotic recombination, nuclear divisions were monitored in a *spo11Δ* background. Cells devoid of *SPO11* do not initiate meiotic recombination and improperly segregate their DNA since they lack attachments between the homologs [2]. Even though the resulting spores are unviable, DNA segregation can be monitored within the cells. Deletion of *SPO11* in *smc6-56*, *smc5-mn*, *nse4-mn* and *nse2-mn* mutants abolished the segregation block (Figure 1F), indicating that the segregation defect in these cells is the result of problems during DSB repair.

To test whether the nuclear division failure was due to break-independent sister entanglements, segregation was examined in cells containing the *smc6-56* mutation in a *spo11Δ spo13Δ* background. *SPO13* is required to prevent biorientation of sister kinetochores at meiosis I, and, in the absence of recombination, *spo13Δ* cells undergo a single meiotic division, segregating sister chromatids to form cells with two viable, diploid spores called dyads [71],[72]. The *spo11Δ spo13Δ smc6-56* mutant segregated its sisters efficiently and formed viable dyads under soft-shift conditions (Figure S3). These data confirm that the segregation block in *smc6* mutants is not due to recombination-independent sister entanglements.

### Sister chromatid cohesion and double-strand break repair are largely unaffected in the *smc6-56* mutant

**Figure 2:**
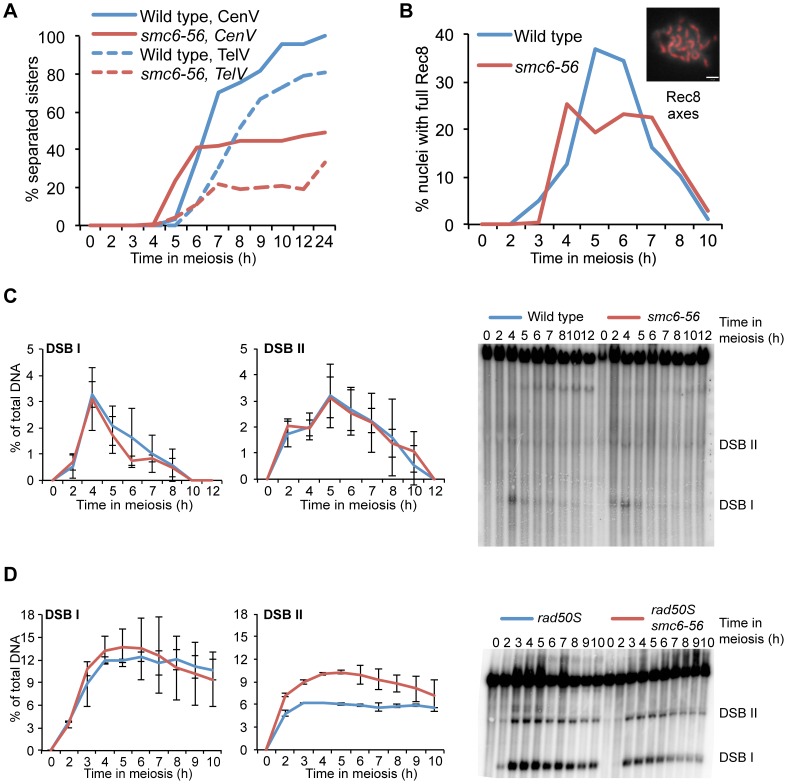
Sister chromatid cohesion, Rec8 dynamics and DSB repair are largely unchanged in *smc6-56* mutants. (**A**) Sister chromatid separation of chromosome V. Percent of sister chromatid separation 35 Kb away from the centromere (CenV) in wild type (CB1197) and *smc6-56* (CB1248) is shown in solid lines. Percent separation 50 Kb away from the right telomere (TelV) in wild type (CB1427) and *smc6-56* (CB1426) indicated in dotted lines. N = 200. (**B**) Percent of cells with full Rec8 protein axes in wild type (CB46) and *smc6-56* (CB1346). Rec8 axes were assessed by detecting epitope-tagged Rec8 (Rec8-3HA) on chromosome spreads using standard immunofluorescence techniques, picture illustrates a cell with full Rec8 axes shown in red, scale bar indicates 2 µm. N = 100. (**C**) DSB repair at the *HIS4LEU2* hotspot on chromosome III in wild type (CB1183) and *smc6-56* (CB1303). The curves represent mean break levels at the two DSB sites at the indicated time points. The Southern blot shown is representative for the three used for quantifications, DSB species were identified according to their size. (**D**) Cumulative DSB levels at the *HIS4LEU2* hotspot in *rad50S* (CB2059) and *rad50S smc6-56* (CB2060). The Southern blot shown is representative for those used to quantify DSBs in the *rad50S* background. Plots in (C) and (D) represent mean ± standard deviation from three independent experiments. All experiments were run under soft-shift conditions.

To further study meiosis in *smc6-56* cells, sister chromatid separation was assessed at sites 35 kb from the centromere and 23 kb from the telomere of chromosome V. These regions were observed using the previously described GFP-tagged Tetracycline repressor/operator (TetR-GFP/Tet-O) system. This system is based on endogenously expressed TetR-GFP, which accumulates at multiple copies of Tet-Os inserted at the chromosomal region of interest, thereby allowing its visualization by fluorescence microscopy [73],[74]. Despite the full segregation block in *smc6-56* cells, no major defect in sister chromatid cohesion or sister chromatid separation was observed at the centromere or telomere of chromosome V (Figure 2A). Final levels of sister chromatid separation did not reach those in wild-type cells, but the results showed that sister chromatids were able to separate within the unsegregated DNA masses in *smc6-56* mutants. This suggests that the Smc5/6 complex has little influence on meiotic sister chromatid cohesion and implies that the chromosomes in *sm6-56* cells are held together by cohesin-independent mechanisms. This notion is further supported by the finding that *smc6-56* cells can separate their sister chromatids in a *spo11Δ spo13Δ* background (Figure S3).

To confirm that remaining cohesin was not the cause of the segregation block in *smc6* mutants, cohesin dynamics were monitored on chromosome spreads using an epitope-tagged version of the meiosis-specific cohesin subunit, Rec8. After being loaded between sister chromatids following DNA replication, Rec8 is removed from chromosome arms at the first nuclear division but maintained at centromeres until MII [75]. If cohesin remains between sister chromatid arms at the first nuclear division, homolog segregation will be blocked due to the inability to resolve COs at the chromosomal level [76]. The *smc6-56* mutant was able to properly localize and remove Rec8 from the chromosome axis (Figure 1E, Figure 2B), leading to the conclusion that the segregation block in this mutant is caused by cohesin-independent chromosome attachments.

To examine the role of the Smc5/6 complex during meiotic break repair, DSBs were monitored at the *HIS4LEU2* hotspot on chromosome III [12] [77] [78]. In this assay, *smc6-56* mutants were able to repair their DSBs efficiently at the two sites analyzed (Figure 2C). To investigate whether *smc6-56* mutants have higher levels of break formation, DSB accumulation was investigated in a *rad50S* background. This mutant cannot resect the ends of the break and accumulates unprocessed DSBs [79]. The *smc6-56 rad50S* mutant had higher levels of breaks at one DSB site but normal levels at the other (Figure 2D). Whole-chromosome break patterns were similar in *smc6-56 rad50S* and *rad50S* on chromosomes III, IV and VI ( and data not shown). These data show that DSB repair and distribution are unchanged in *smc6* mutants but that overall DSB levels may be higher, at least at specific sites.

### Functional *SMC6* is required during joint molecule resolution

**Figure 3:**
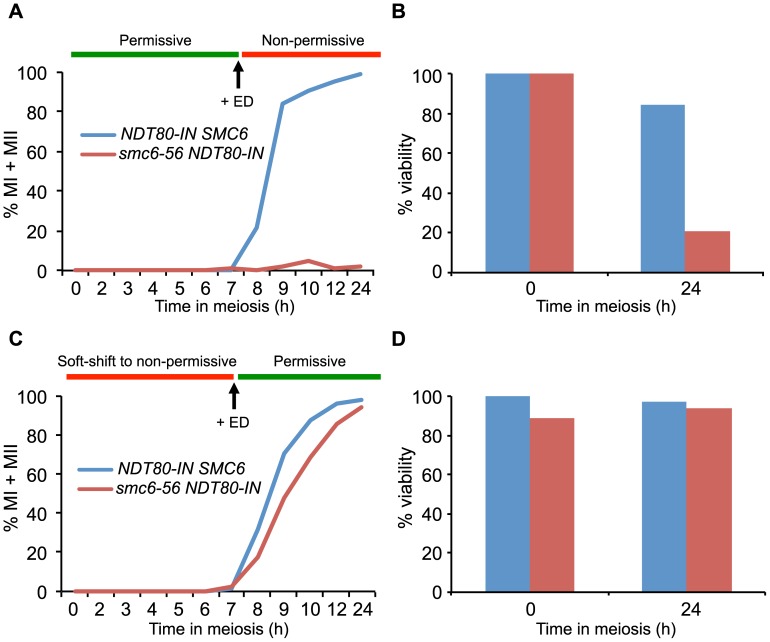
The Smc5/6 complex performs its most critical functions at the time of joint molecule resolution. Meiotic progression and cell viability were determined following temperature shifts in strains carrying an inducible *NDT80* allele (*NDT80-IN*) under the control of estradiol (ED). Meiotic progression given as percent of MI+MII cells in *NDT80-IN SMC6* (CB1753) and *NDT80-IN smc6-56* (CB1723) at the indicated time points. N = 200. For viability assessment, cells were collected at the indicated time points, sonicated briefly, diluted to the desired concentration, spread onto YPD plates and grown at permissive temperature for 3 days. Viability is given in percent as determined by the number of colony-forming units divided by the total number of cells plated. (**A**) Cells were accumulated in an *ndt80* arrest at permissive temperature until 7 h when 1 µM β-estradiol (+ED, arrow) was added and cultures were shifted to non-permissive temperature. (**B**) Cell viability for cells undergoing meiosis under the same conditions described for (A) at 0 h and 24 h after meiotic induction. (**C**) Cells were accumulated in *ndt80* arrest under soft-shift conditions at non-permissive temperature until 7 h when 1 µM β-estradiol (+ED, arrow) was added and cultures were shifted to permissive temperature. (**D**) Cell viability for cells undergoing meiosis under the same conditions described for (C) at 0 h and 24 h after meiotic induction.

The segregation block in the *smc5/6* mutants is reminiscent of that observed in mutants that are unable to resolve JMs [8],[17],[80],[81]. Factors that promote JM resolution and subsequent prophase exit are activated by the transcription factor Ndt80. In the absence of *NDT80*, cells accumulate in late prophase with unresolved JMs [82],[82]. To initially assess if the Smc5/6 complex also plays a role during JM resolution, cells in which expression of *NDT80* is controlled by an estradiol-inducible promoter (*NDT80-IN*) were utilized [83]. Combining the *smc6-56* allele with *NDT80-IN* allowed the control of Smc6 activity by temperature shifts carried out concurrently to *NDT80* induction. The *smc6-56* cells were unable to segregate their DNA when taken into the *ndt80* arrest at permissive temperature and released at non-permissive temperature (Figure 3A). This was not due to incomplete arrest at the time of temperature upshift and *NDT80* induction, since *smc6-56* cells kept in the *ndt80* block three hours longer before shifting to non-permissive temperature showed the same segregation block (data not shown). At the final time point after release into non-permissive temperature after arrest at permissive temperature, *smc6-56* cells were largely inviable (Figure 3B). This suggests that the structures which block segregation are also lethal to the cells. If the *smc6-56* mutant instead underwent the *ndt80* arrest under soft-shift conditions and was shifted to permissive temperature during release, meiotic divisions were restored and cells completed both MI and MII with wild-type kinetics (Figure 3C). These cells were also viable at the final time point (Figure 3D). These data imply that *SMC6* is most critical during JM resolution, and suggest that unresolved JMs are the cause of the segregation block and inviablilty in *smc6* mutants.

### The *smc6-56* mutant accumulates unresolved joint molecules

**Figure 4:**
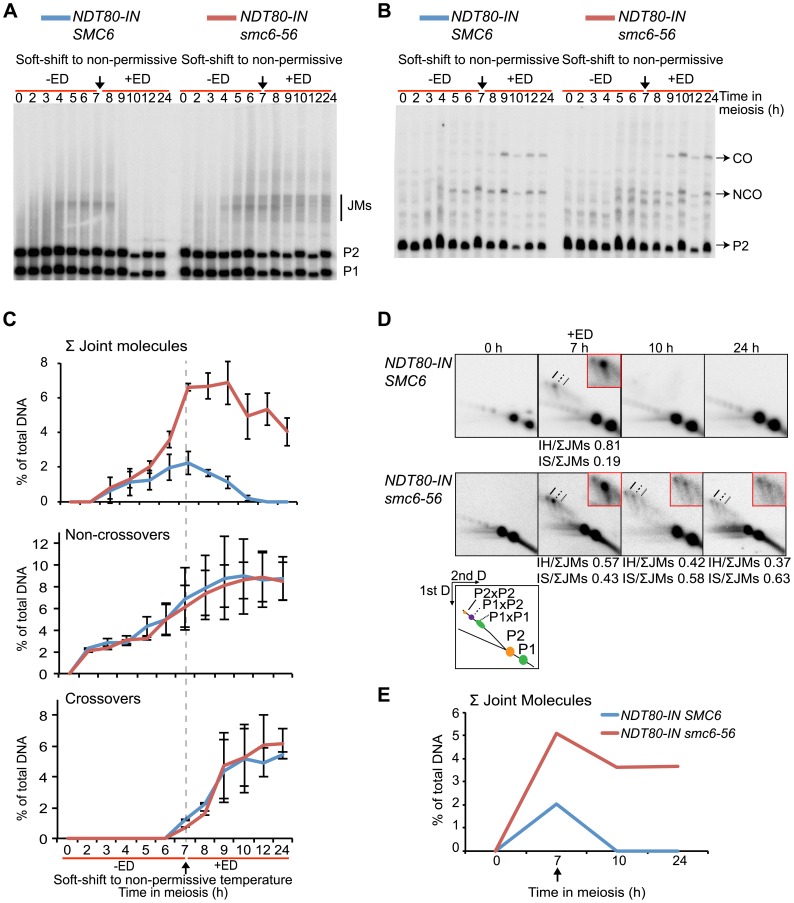
The *smc6-56* mutant accumulates unresolved joint molecules but forms normal levels of recombination products. Analysis of recombination measured at the ectopic *URA3-ARG4* interval on chromosome III (Figure S5) [84] in strains containing the estradiol-inducible *NDT80* allele (*NDT80-IN*) under soft-shift conditions. *NDT80-IN* was induced at 7 h with 1 µM β-estradiol (+ED, arrow). Blue curves indicate *NDT80-IN SMC6* (CB2096); red *NDT80-IN smc6-56* (CB2097). (**A**) Representative Southern blot used to detect JMs after digesting with *XmnI* and probing for a region in *ARG4*. P1, P2 and JM regions based on expected sizes. (**B**) Representative Southern blot used to detect CO/NCO products at the same interval after digesting with *XhoI/EcoRI* and probing for *HIS4* sequences. P2, CO and NCO regions based on expected sizes. (**C**) Quantifications of total JM levels, NCOs and COs in percent of total DNA from blots illustrated in (A) and (B). Dotted line and arrow indicate time of *NDT80-IN* induction. Plots represent mean ± standard deviation from three independent experiments. (**D**) Two-dimensional analysis of JM species. DNA from *NDT80-IN SMC6* and *NDT80-IN smc6-56* undergoing meiosis under soft-shift conditions was isolated and subjected to two-dimensional electrophoresis as described in Materials and Methods. The interpretive panel shows the assumed identity of JM species after probing for a region which recognizes both homologs. JMs between P1×P1 are the result of random breaks and are therefore expected to give a broader signal than P2×P2 and P2×P1. Dashed line, P2×P2 IS-JM; solid line, P2×P1 IH-JM; dotted line, P1×P1 IS-JM. The enlarged panels are enhanced images of the JM region from the designated time point. Ratio of IH-JMs/ΣJMs and IS-JMs/ΣJMs given below relevant images and were calculated as described in Figure S9. (**E**) Quantification of total JMs in percent of total DNA from the gels represented in (D). Arrow denotes *NDT80* induction. Curves represent one experiment, with similar results from a second independent experiment presented in Figure S7A.

To assess whether JMs accumulate in *smc6-56* mutants, recombination was examined at the molecular level at the ectopic *URA3-ARG4* locus on chromosome III, which allows the detection of JMs in the form of dHJs using one-dimensional (1D) gel electrophoresis (Figure S5) [84]–[86]. This hotspot was used in combination with *NDT80-IN* under soft-shift conditions to test the hypothesis that the segregation block in *smc6-56* cells is caused by the accumulation of unresolved JMs. The *smc6-56* mutant accumulated a ≈3-fold higher number of JMs than the *SMC6* strain (6.6% vs. 2.2% at 7 h) prior to *NDT80* induction (Figure 4A–C). Following induction, approximately two-thirds of JMs in the *smc6-56* mutant remained unresolved after 24 h. In spite of unresolved recombination intermediates, NCOs and COs accumulated at the same time and level in the *smc6* mutant as compared to the *SMC6* strain (Figure 4B,C). Recombination products were also observed at wild-type levels at the *HIS4LEU2* hotspot in the *smc6-56* mutant (data not shown).

To better identify JM species, native-native two-dimensional (2D) gel electrophoresis was utilized at the *URA3-ARG4* locus in combination with the *NDT80-IN* system. This method of electrophoresis separates JMs by size in the first dimension and by shape plus size in the second dimension [87]. Prior to *NDT80* induction, *SMC6* cells accumulated a strong JM spot corresponding to the predicted size for IH-JMs (P1×P2) (Figure 4D, 7 h). This spot was flanked by two weaker regions: a slower-migrating spot predicted to be IS-JMs from P2 (P2×P2) and a faster-migrating, less defined spot corresponding to IS-JMs from P1 (P1×P1) (Figure 4D). To verify the identity of these flanking spots as IS-JMs, recombination was examined in cells lacking the axial element protein Hop1, in which the sister chromatid is preferred over the homolog as a repair template [42],[88]. As anticipated, the *hop1Δ* mutant lacked the middle spot corresponding to IH-JMs and acquired the two outer spots predicted for IS-JMs, with P2×P2 being the dominating IS species (Figure S6A). The indistinctness of the P1×P1 spot is due to the fact that the DSB hotspots at this locus are only located on the P2 homolog, directing the majority of inter-sister repair to this set of sister chromatids (Figure S5).

Similar to the results obtained from the 1D gels, levels of total JMs from the 2D gels were approximately 2.5-fold higher in the *smc6-56* mutant than in the *SMC6* strain prior to *NDT80* induction (Figure 4D–E). A homolog-specific probe identified many of these JMs as IS-JMs in the *smc6-56* mutant (Figure S6B). Though some JMs in the *smc6-56* mutant were resolved following release, about two-thirds of the total persisted at the final time point (Figure 4E). Further examination of JM composition revealed that *smc6-56* cells formed JMs composed of a higher ratio of IS-JMs compared to *SMC6* cells in the *ndt80* arrest (Figure 4D 7 h, 0.43 vs. 0.19 of total). After *NDT80* induction, the ratio of IS-JMs to total JMs increased to over 0.6 in *smc6-56* cells, though some IH-JMs persisted as well. Similar results were obtained in an independent experiment (Figure S7A). These data indicate that *SMC6* prevents the formation of excess JMs and facilitates the resolution of JMs; especially those formed between sister chromatids.

### *SMC6* is required for the resolution of joint molecules

**Figure 5:**
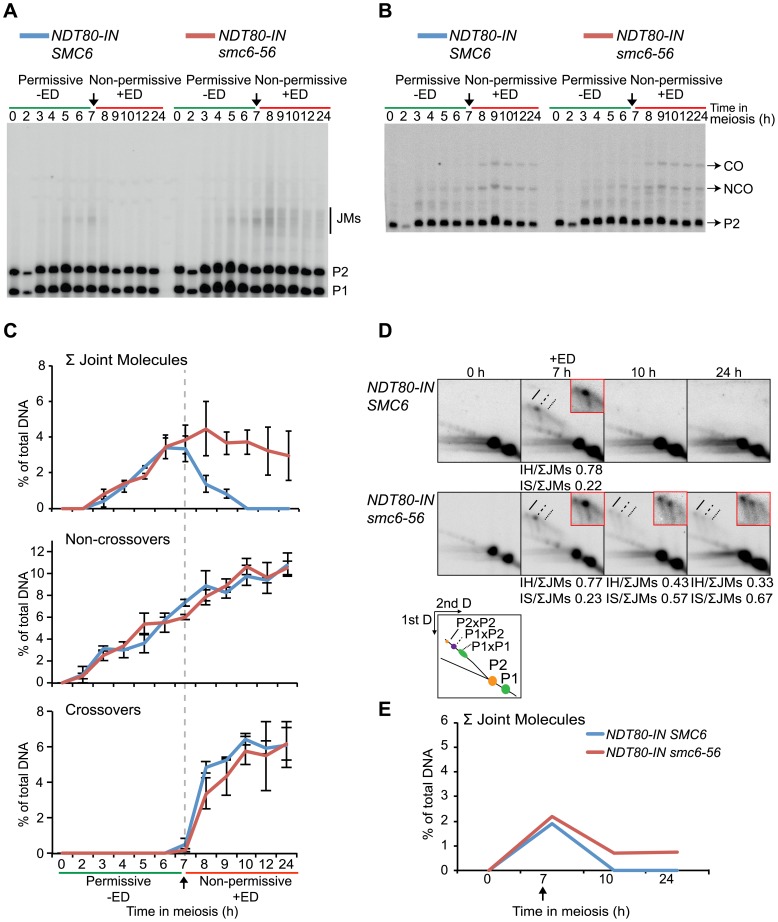
Smc6 protein function is required to resolve a subset of joint molecules. Cells were accumulated in an *ndt80* arrest at permissive temperature (green lines, −ED) until *NDT80* induction when cultures were shifted to non-permissive temperature (red lines, +ED) at 7 h. Arrows indicate addition of 1 µM β-estradiol. Blue curves indicate *NDT80-IN SMC6* (CB2096) and red curves indicate *NDT80-IN smc6-56* (CB2097). One-dimensional JM and CO/NCO detection and two-dimensional JM analysis were performed using the same locus and techniques described in Figure 4. (**A**) Representative Southern blot used to detect joint molecules. (**B**) Representative Southern blot used to detect CO/NCO species. (**C**) Quantifications of total JM levels, NCO and CO products from blots in (A) and (B), respectively. Dotted line and arrow indicate time of *NDT80* induction. Plots represent mean ± standard deviation from three independent experiments. (**D**) Identification of JM species using two-dimensional electrophoresis on *NDT80-IN SMC6* and *NDT80-IN smc6-56* undergoing *NDT80* arrest at permissive temperature and release at non-permissive temperature. The enlarged panels are enhanced images of the JM region from the designated time point. Ratio of IH-JMs/ΣJMs and IS-JMs/ΣJMs given below relevant images and were calculated as described in Figure S9. (**E**) Quantification of total JMs in percent of total DNA from the gels represented in (D). Arrow denotes *NDT80-IN* induction. Curves represent a single experiment, with similar results from a second independent experiment shown in Figure S7B.

To test if *SMC6* is needed for JM resolution, JMs were examined under conditions when the Smc6 protein was functional during the *ndt80*-mediated arrest and then made non-functional during release. In this situation, the *smc6-56* mutant was inviable and unable to segregate its DNA (Figure 3A–B). JM formation was normal when *smc6-56* cells were arrested at permissive temperature (Figure 5A–E). When shifted to non-permissive temperature at the time of *NDT80* induction, JMs were not fully resolved, and no significant decrease in CO or NCO levels was detected (Figure 5A–E). Possible reasons for the counter-intuitive finding that IH-JMs persist without a detectable decrease in CO formation are considered in the discussion. Upon closer examination, the ratio of IS-JMs out of total JMs at the final time point is increased to 0.67 from its ratio of 0.23 at the time of *ndt80* release (Figure 5D). IH-JMs remained as well, but some were apparently resolved, as reflected in the decreased IH-JM ratio and formation of COs at later time points. Similar results were found in an independent experiment (Figure S7B). Together these results implicate a role for *SMC6* in the resolution of IS-JMs and, to a lesser extent, IH-JMs that form under normal conditions.

### Joint molecules accumulated in the absence of *SMC6* function are resolved after restoration of Smc6 activity

**Figure 6:**
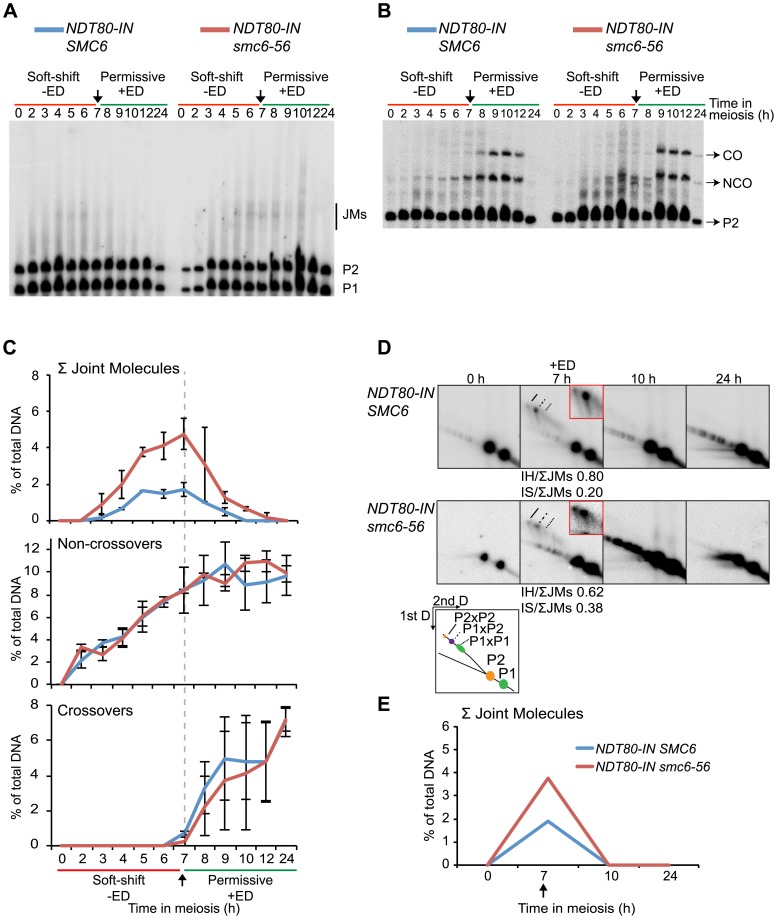
Joint molecules accumulated without functional Smc6 protein can be resolved when its function is restored during resolution. Cells were accumulated in an *ndt80* arrest with soft-shift to non-permissive temperature (red lines, −ED). *NDT80* was induced concurrently with the shift to permissive temperature (green lines, +ED) at 7 h. Arrows indicate addition of 1 µM β-estradiol. Blue curves indicate *NDT80-IN SMC6* (CB2096) and red curves indicate *NDT80-IN smc6-56* (CB2097). One-dimensional JM and CO/NCO detection and two-dimensional JM analysis were done using the same locus and techniques described in Figure 4. (**A**) Representative Southern blot used to detect joint molecules. (**B**) Representative Southern blot used to detect CO/NCO species. (**C**) Quantifications of total JM levels, NCO and CO products from blots in (A) and (B), respectively. Dotted line and arrow indicate time of *NDT80* induction. Plots represent mean ± standard deviation from three independent experiments. (**D**) Identification of JM species using two-dimensional electrophoresis on *NDT80-IN SMC6* and *NDT80-IN smc6-56* undergoing *NDT80* arrest at non-permissive temperature and release at permissive temperature. The enlarged panels are enhanced images of the JM region from the designated time point. Ratio of IH-JMs/ΣJMs and IS-JMs/ΣJMs given below relevant images and were calculated as described in Figure S9. (**E**) Quantification of total JMs in percent of total DNA from the gels represented in (D). Arrow denotes *NDT80-IN* induction. Curves represent one experiment, with similar results from a second independent experiment shown in Figure S7C.

When Smc6 is non-functional during *ndt80* arrest but functional during release, cells successively completed nuclear divisions (Figure 3C) and formed normal levels of CO and NCO products (Figure 6A–C). Prior to *NDT80* induction, the *smc6-56* mutant formed ≈3-fold higher total JM levels (Figure 6C, E). At this time point, the ratio of IS-JMs to the total on the 2D gels was 0.62 in the *smc6* mutant, compared to 0.20 in the *SMC6* strain. When Smc6 function was restored at the time of *NDT80* induction, all JMs were resolved (Figure 6A,C,D,E). Similar results were obtained from 2D gels from an independent experiment (Figure S7C). This supports the notion that the rescue in nuclear divisions is due to the restoration of JM resolution. The JMs that accumulated in the absence of *SMC6* function were not lethal, as viability was also restored when the cells were shifted to permissive temperature (Figure 3D). These results show that all JMs formed without functional Smc6 can properly be resolved if Smc6 function is restored during JM resolution.

### Smc6 localization on meiotic chromosomes depends on cohesin

**Figure 7:**
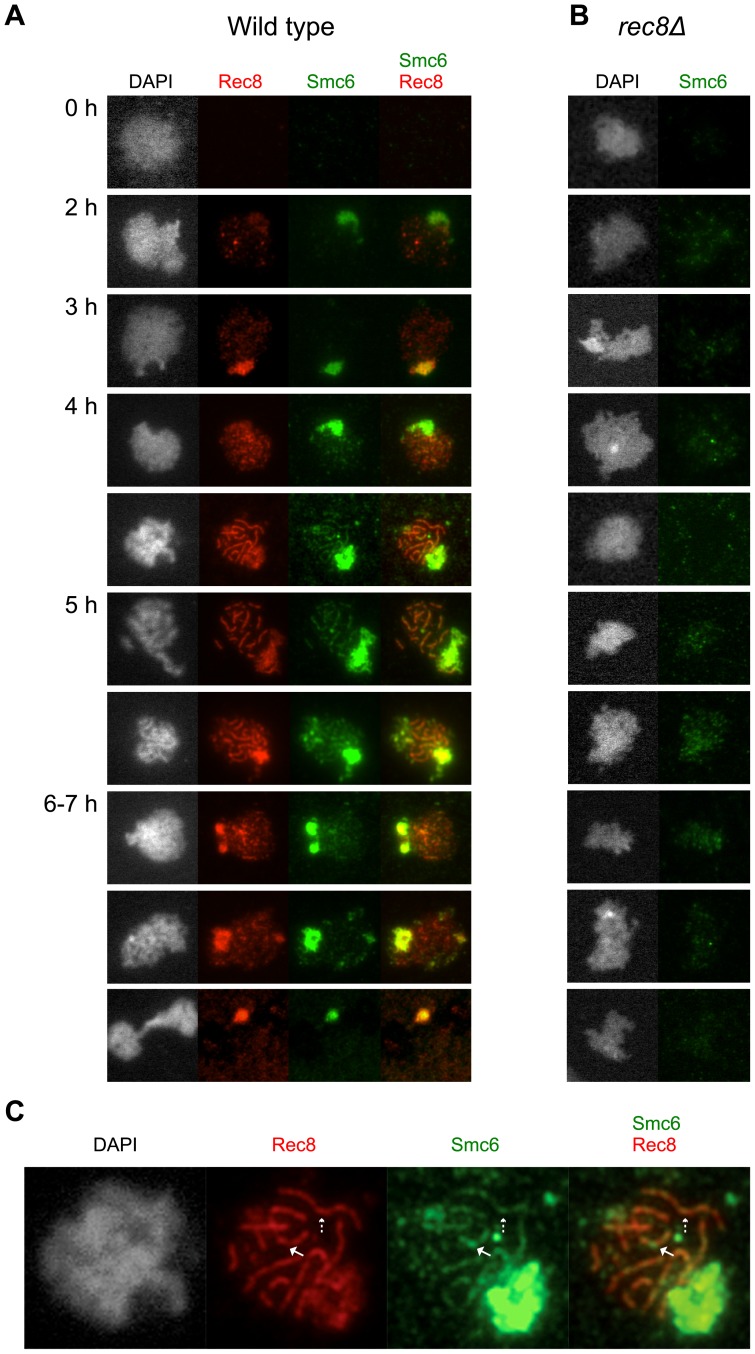
The Smc6 protein localizes to meiotic chromosomes during prophase in a cohesin- dependent manner. Cells were isolated at the indicated time points and surface-spread to detect epitope-tagged proteins Smc6 (Smc6-13Myc, green) and Rec8 (Rec8-3HA, red) using standard immunofluorescence techniques. DNA visualized with DAPI shown in grey. (**A**) Wild type (CB1361). (**B**) *rec8Δ* (CB1411). (**C**) Enlarged image of a representative cell from the wild type (CB1361) at the 4 h time point. Solid arrows indicate an Smc6 site at a weaker-staining Rec8 site. Dashed arrows indicate a strong Smc6 site on top of a strong Rec8 site.

To gain additional insights into the function of the Smc5/6 complex during meiotic recombination, an N-terminal epitope-tagged version of Smc6 was used to analyze the complex's binding on chromosome spreads using immunofluorescence. The tagged version of *SMC6* was fully functional and neither impeded events during meiotic prophase nor delayed meiotic segregation (data not shown). Smc6-Myc appeared on chromosomes during early prophase around the time of Rec8 foci formation (Figure 7A). When the Rec8 axis began to organize, Smc6's binding became more profuse and formed an axis-like structure. On full-length axes, Smc6 localized at regions with weaker Rec8 signals as well as at sites with more profuse Rec8 signals (Figure 7C, solid and dashed arrows, respectively). Consistent with results from a previous study [60], Smc6 bound abundantly to the rDNA, seen by the brightly staining Smc6 region (Figure 7A, green) corresponding to weak DAPI staining. After late prophase, the Smc6-Myc signal became diffuse and disappeared prior to MI. Removing cohesin seemed to reduce the amount of Smc6 foci and eliminated the axis-like pattern of Smc6 (Figure 7B). Western blotting revealed that Smc6 protein levels were similar in the wild type and in the *rec8Δ* mutant, indicating that the diminished levels of Smc6 binding were not due to decreased Smc6 protein levels in this cohesin mutant (Figure S8). This demonstrates that cohesin is required for the proper organization of Smc6 foci on chromosomes and suggests that localization of the Smc5/6 complex is influenced by meiotic axis structure and/or the presence of sister chromatid cohesion.

## Discussion

**Figure 8:**
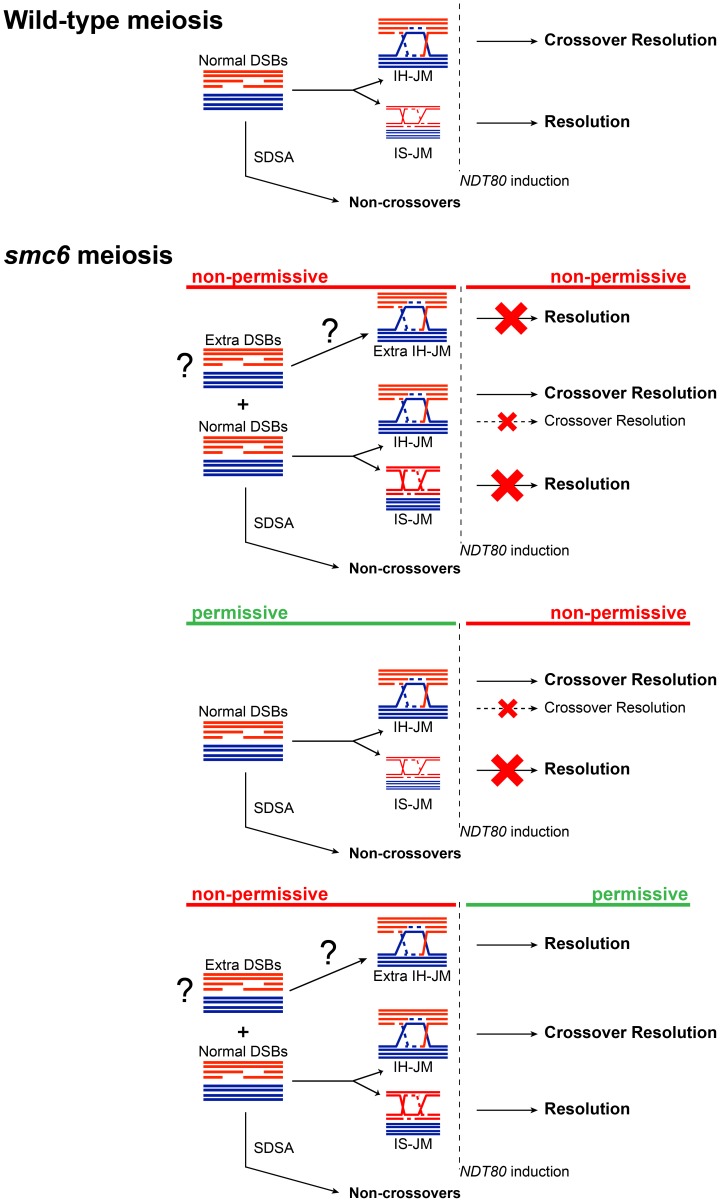
Model for the role of the Smc5/6 complex during meiosis. Schematic diagram depicting the result of having non-functional Smc6 protein before and/or after *NDT80* induction as indicated (red = non-permissive/non-functional, green = permissive/functional) and described in the text.

The results presented here suggest that the Smc5/6 complex prevents excessive JM formation and aids in JM resolution during meiosis. This resolution function is particularly critical for JM intermediates formed between sister chromatids. Similar to other mutants defective in JM processing, *smc5/6* mutants experience a recombination-dependent segregation block without halting meiotic progression [8],[17],[80],[81]. This could indicate that Smc6 works together with established resolution pathways, such as those mediated by the ZMMs or Sgs1/Mus81-Mms4. Unlike those mutants, however, the *smc6-56* mutant does not lead to any detectable decrease in CO or NCO levels, suggesting that it works predominately outside of canonical meiotic recombination pathways. Given the normal levels of COs and NCOs, and the nature of the persisting JMs, we propose that in the absence of Smc6, cells accumulate primarily IS-JMs, but also a subset of IH-JMs (Figure 8).

When JM formation occurs without Smc6 function, overall JM levels are 2.5–3-fold higher than in cells with functional Smc6 (Figure 4C,E, Figure 6C,E). The ratio of IS-JMs to total JMs in the *smc6-56* mutant is twice that in *SMC6* cells (Figure 4D, Figure 6D). Although IH-JMs are still the dominating species, the absence of Smc6 diminishes the IH-bias slightly. This may be due to higher levels if IS recombination in the *smc6-56* mutant or due to an accumulation of normally transient IS-JM intermediates that cannot be resolved without functional Smc6. When *NDT80* is induced in the continued absence of Smc6 function, about three-fourths of total JMs persist and the IS-JM ratio increases further, though some IH-JMs also persist (Figure 4C,D). Surprisingly, CO formation is normal in spite of persisting IH-JMs. One explanation for this could be that these lingering IH-JMs are not significant enough in number to cause a detectable decrease in CO levels (Figure 8). As an alternative, extra IH-JMs could come from additional recombination-initiating events, which has been shown to occur in some mutants [11]. The *smc6-56* mutant does form slightly higher numbers of DSBs at one break site in the *rad50S* background (Figure 2D, DSBII). If this also occurs at other sites, it may account for some of the increase in the levels of recombination in the *smc6* mutant.

More recombination-initiating events would also explain the IH-JMs that are never resolved, despite normal CO levels, when Smc6 is non-functional from the time of meiotic induction (Figure 4A–D). This suggests that cells lacking functional Smc6 accumulate recombination intermediates that will require Smc6 for their resolution. In line with this hypothesis, when cells lacking Smc6 function during JM formation are released from the *ndt80* arrest in the *presence* of functional Smc6, all JMs are resolved, despite the higher ratio of IS-JMs at the time of release and higher overall JM levels (Figure 6). COs and NCOs are formed efficiently (Figure 6B,C), and DNA segregation and viability is rescued (Figure 3C,D). This indicates that the JMs formed in the mutant can be properly resolved when Smc6 function is restored (Figure 8). This reversible phenotype is similar to what has been observed for the inter-sister recombination intermediates which accumulate in mitotic cells lacking Smc6 function [89].

Finally, when Smc6 is functional during JM formation in an *ndt80*-mediated arrest, both the levels and ratios of JMs are normal (Figure 5A,C,D,E). Upon release into conditions that render Smc6 non-functional, however, three-fourths of total JMs persist and cells are unable to segregate their DNA (Figure 5C, Figure 3A). The remaining JMs are composed of both IS-JMs and IH-JMs, but the ratio of IS-JMs increases upon shift to non-permissive temperature (Figure 5D). A fraction of IH-JMs is resolved, as reflected in the efficient formation of COs and decrease in the IH-JM ratio (Figure 5A–D). One explanation for why these unresolved IH-JMs do not lead to a detectable decrease in CO formation is that they contribute to a very small portion of total CO levels not distinguishable in the assays used here (Figure 8). NCO formation is also normal, indicating that the remaining JMs are not caused by converted SDSA events (Figure 5B,C). These data suggest that a subset of IH-JMs that form under normal conditions require Smc6 for their resolution, while nearly all IS-JMs seem to rely on Smc6 for their resolution (Figure 8). This finding correlates with the role of the Smc5/6 complex in the resolution of sister chromatid intermediates during homologous recombination during mitosis in yeast [53] and in germ line cells in *C. elegans* [57].

Even though the presented evidence suggests a role for the Smc5/6 complex outside of canonical recombination pathways, the possibility that it works together with other recombination pathways cannot be ruled out (Eva Hoffman, personal communication; Franz Klein, personal communication). In a wild-type meiosis, almost all NCOs are derived from the SDSA pathway and do not resolve via a JM intermediate [11],[12]. In contrast, in cells lacking *SGS1*, what should have been SDSA events are instead stabilized and transformed into dHJs [8]. These are later resolved into both NCO and CO products, thereby delaying the timing of NCO formation until JM resolution is induced [8]. With that in mind, the additional JMs in the *smc6-56* mutant presented here could come from the conversion of NCO-forming SDSA events into JM intermediates. However, the *smc6-56* mutant is not defective in the timing of NCO formation under non-permissive conditions, and forms most of its NCOs prior to *NDT80* induction (Figure 4B,C, Figure 6B,C). Final NCO levels are also normal, indicating that the JMs that remain in the *smc6-56* mutant are not derived from the conversion of SDSA events into unresolvable JMs.

Here we show that the *smc6-56* mutant is able to establish sister chromatid cohesion and efficiently localize and remove cohesin from chromosomes (Figure 2B, Figure 1C). Mutants in other subunits of the Smc5/6 complex have been reported to inhibit full removal of meiotic cohesin (Eva Hoffman, personal communication). One explanation for this discrepancy could be that some components of the Smc5/6 complex work in different pathways. The segregation block in an *smc5-mn* mutant is also reported to be partially rescued when Rec8 is artificially removed from chromosomes (Eva Hoffman, personal communication). While this result could point towards a function for Smc5 in cohesin removal, it could also support the notion that the Smc5/6 complex is needed to resolve IS recombination intermediates. Removing sister chromatid cohesion reduces the likelihood of IS repair, thereby decreasing the level of IS recombination and allowing some *smc5-mn* cells to segregate their chromosomes. While the *smc6-56* mutant can separate some of its sister chromatids at the telomere and centromere on chromosome V, this segregation is not complete (Figure 2A). This is not due to an abnormal version of cohesion, as cells forced to undergo a mitosis-like division in the absence of recombination can separate their sister chromatids (Figure S3). Instead, the sisters are most likely held together by DNA attachments and not cohesin, as illustrated by data showing that the *smc6-56* mutant accumulates unresolved JMs between sister chromatids. In addition, the centromeric and telomeric regions have been suggested to be hotspots for meiotic inter-sister repair [90], which could explain why less than half of the sister chromatids are able to separate at these regions despite cohesin removal in *smc6-56* cells.

Smc6 localizes to meiotic chromosomes as well as to the rDNA (Figure 7A). Preliminary evidence suggests that its binding pattern may reveal its precise localization on the meiotic axis, i.e. whether it sits at or between Rec8 sites (Figure 7C). Indeed, abolishing sister chromatid cohesion by removing Rec8 reduces the binding of Smc6 to chromosomes, suggesting that cohesin may guide Smc6 localization (Figure 7B). It is possible that the absence of sister chromatid cohesion reduces the likelihood of inter-sister recombination, which in turn diminishes the loading of Smc6. Alternatively, or in addition, the stable association of Smc6 to chromosomes may require proper axis formation and/or cohesion as such. Deletion of *REC8* only diminishes the binding of Smc6, indicating that other factors dictate the loading of Smc6 to meiotic chromosomes. It will be interesting to learn what role the Smc5/6 complex plays in meiotic chromatin organization in order to gain further insights into its role during recombination.

In conclusion, this study identifies a crucial role for the Smc5/6 complex in processing of recombination intermediates during meiotic recombination. Mutants in the Smc5/6 complex acquire high levels of recombination intermediates between homologs and sister chromatids. The majority of IH-JMs are resolved, as reflected by the decreased ratio of IH-JMs at the final time points and the normal level of COs. IS-JMs, on the other hand, seem to depend on the function of Smc6 for their resolution. We therefore propose that the main impediment to homolog segregation in *smc5/6* mutants is unresolved linkages between sister chromatids, though some homolog attachments contribute to the segregation defect as well.

## Materials and Methods

### Yeast strains and methods

Strains used for this investigation are derivatives of SK1 [91] and are shown in Table S1. Gene deletions and C-terminal epitope tags were introduced using standard methods [92]. The *smc6-56* allele was integrated at its endogenous locus and contains three point mutations in the coil-coil region of the protein which render it temperature-sensitive [61]. Meiotic nulls for *SMC5*, *NSE4* and *NSE2* were made by replacing the endogenous promoters with the *CLB2* promoter using one-step gene replacement [62]. The *NDT80-IN* strains have been described [82],[83].

### Sporulation

Liquid media, pre-sporulation and sporulation conditions were done using SPS media according to previously described methods [93]. Cultures were grown with vigorous shaking in baffled flasks at least ten times larger than the culture volume to achieve optimum synchrony. Permissive temperature was defined as 25°C, non-permissive temperature was 33°C. For the soft-shift setup, pre-sporulation plates and cultures were grown at 25°C. Once shifted to sporulation media, the cells were grown for 2.5 hours at 25°C before raising the temperature to 33°C. All experiments were performed at least twice with results similar to those presented in the figures here. Expression of *NDT80-IN* was induced by the addition of β-estradiol at a final concentration of 1 µM 7 h after meiotic induction.

### Molecular analyses

The *HIS4LEU2* locus used for DSB analysis has been described [6],[78]. The ectopic locus on chromosome III used for 1D and 2D JM analyses as well as CO/NCO detection is illustrated in Figure S5 and has been described [11] [11]. For native-native two-dimensional gel electrophoresis, psoralen cross-linked DNA was extracted from meiotic cultures as described in [94] and references therein. After digesting with *XmnI*, DNA samples were run on a 0.4% SeaKem GTG agarose gel (Lonza) lacking ethidium bromide in 1X TBE (90 mM Tris-borate, 2 mM EDTA pH 8) at 1 V/cm for 24 hours at room temperature. Gels were stained for 10 minutes in 1X TBE containing 0.3 µg/mL ethidium bromide, and lanes were excised and laid perpendicular to the direction of current for the second dimension. The gel for the second dimension, 0.8% SeaKem GTG agarose (Lonza) in 1X TBE plus 0.3 µg/mL ethidium bromide, was cast around the gel slices and allowed to harden. Electrophoresis in the second dimension was carried out in 4°C for 6 hours at 4 V/cm in 1X TBE containing 0.3 µg/mL ethidium bromide with pumping from the cathode to the anode. Gels were subjected to Southern blot analysis and probed with *ARG4* coding sequences (+165 to +1413). DNA preparation and one-dimensional electrophoresis for JM assays were done as described using conditions that stabilize JM intermediates [95]. JMs were analyzed using *XmnI* digests probed with *ARG4* coding sequences (+165 to +1413, argD), COs/NCOs were analyzed using *XhoI/EcoRI* double digests probed with *HIS4* coding sequences (+538 to +718, hisU). DNA was transferred to nylon membranes via downward capillary transfer using standard techniques. After cross-linking the DNA, the membranes were pre-hybridized in Church buffer (1% w/v BSA, 1 mM EDTA, 0.5 M phosphate buffer, 7% w/v SDS) for approximately 4 hours at 65°C and hybridized with the radioactively labeled probe overnight at 65°C. After washing, signals were detected on an imaging plate with a FLA-7000 image reader and quantified using Multi Gauge software, all from Fujifilm.

Quantifications of 1D and 2D gels were done using Multi Gauge software by selecting equivalent regions of interest, including one to measure the background of the region. Sizes of expected products were determined using molecular weight standards. The signal was corrected for background and divided by the sum of the measured region and the standard region (in all cases, the parental region). Similar blots were treated equally; for instance, regions of interest used to measure CO/NCO species were the same size for each blot. Further details regarding quantifications of JM species from 2D gels are given in Figure S9. All experiments were performed at least twice with results similar to those presented.

### Cytology and immunofluorescence

Nuclear morphology was scored by DAPI (4′,6-diamidino-2-phenylindole) staining of ethanol-fixed cells using standard protocols. *In situ* immuno-staining of fixed whole cells for microtubule detection was performed using conventional techniques with a monoclonal mouse anti-alpha Tubulin antibody (*DM1A*, Abcam) at a 1∶1000 dilution. Stained slides were mounted and DAPI-stained using ProLong Gold (Invitrogen). Meiotic spreading was done on SuperFrost Plus slides according to the protocol previously described [96] with the exception that 5% Lipsol was used as a detergent. A 1∶500 dilution of rabbit-anti-Zip1 (gift from K. Schmekel) was used to detect Zip1. Smc6-13Myc was detected using 1∶200 mouse-anti-Myc (Invitrogen) and Rec8-3HA was detected using 1∶200 rat-anti-HA (Roche). Stained slides were mounted and DAPI-stained using ProLong Gold (Invitrogen). Image acquisition of a single focal plane was done in Volocity (Perkin Elmer) with a Leica confocal microscope. Image processing and analysis was carried out in Volocity.

Additional methods describing results shown in supporting figures can be found in Text S1.

## Supporting Information

Figure S1 Schematic representation of meiotic recombination. Meiotic recombination is initiated by Spo11-catalyzed DNA double-strand breaks (DSBs). Spo11 is removed from the DNA in the form of Spo11-oligonucleotide complexes, allowing the 5′ ends of the DSB to be resected to generate 3′ single-stranded overhangs coated by Rad51 and Dmc1 (not shown) that can invade a homologous strand for repair. Strand invasion gives rise to a D-loop, forming an initial joint molecule (JM) intermediate. Following stabilization and DNA synthesis, the initial JM gives rise to another transient JM species called the single-end invasion (SEI). (**A**) The SEI can be quickly dissociated to re-ligate the newly synthesized DNA end to the complementary free break end in a process called synthesis-dependent strand annealing (SDSA). Additional DNA synthesis and ligation yields a mature non-crossover product. (**B**) Alternatively, the SEI can be stabilized to facilitate capture of the second 3′ DSB end via engagement of the intact homologous strand. Further processing yields gives rise to a stable JM intermediate known as a double-Holliday junction (dHJ). (**C**) Endonuclease-mediated resolution of the dHJ yields primarily crossover products.
(TIF)

Figure S2 Segregation, viability and FACS profiles from wild-type and *smc6-56* strains. (**A**) Meiotic progression and viability of wild type (CB1017) and *smc6-56* (CB1032) at permissive temperature (25°C). Progression indicated as percent of MI+MII cells observed via DAPI staining of fixed whole cells, N = 200. Spore viability determined after dissection of 72 spores following sporulation for 3 days at permissive temperature. (**B**) Meiotic progression in wild type (blue curves, top) and *smc6-56* (red curves, bottom) at non-permissive temperature (33°C). The *smc6-56* mutant does not divide its nuclei and instead forms cells containing one DNA mass outside of four spores as described in Figure 1 but also has a population of cells that remain mononucleate when kept at non-permissive temperature from the time of transfer into meiotic media. N = 200. (**C**) FACS profiles from wild type (blue) and *smc6-56* (red) undergoing meiosis under soft-shift conditions. Cultures were kept at permissive temperature (25°C) until 2.5 hours after meiotic induction (red asterisk) when the majority of the cells in the wild type and mutant had completed replication and the temperature was raised to non-permissive (33°C).
(TIF)

Figure S3 Segregation and spore viability in a *spo11Δ spo13Δ* background. (**A**) Percent dyad formation in *spo11Δ spo13Δ* (CB1466) and *smc6-56 spo11Δ spo13Δ* (CB1465) undergoing meiosis under soft-shift conditions. N = 200. (**B**) Spore viability after dissection of 36 spores for *spo11Δ spo13Δ* (CB1466) and *smc6-56 spo11Δ spo13Δ* (CB1465) after sporulation for three days at non-permissive temperature. Spores were grown for 3 days at permissive temperature.
(TIF)

Figure S4 Whole-chromosome break pattern of chromosome IV in a *rad50S* background. Analysis of whole-chromosome break pattern for chromosome IV for *rad50S* (CB58) and *rad50S smc6-56* (CB1360) strains undergoing meiosis under soft-shift conditions. At the indicated time points, cells were isolated and treated for DNA extraction and subsequent pulse-field gel electrophoresis as described in Text S1.
(TIF)

Figure S5 The *URA3-ARG4* recombination hotspot on chromosome III. Figure is adapted from that shown in [84]. The *URA3-ARG4* construct is inserted at *LEU2* on one homolog (P1) and at *HIS4* on the other homolog (P2). P2 contains an *EcoRI* site-containing palindrome (indicated by the grey circle) in the *ARG4* sequence, denoted *arg4-EcPal*. The restriction sites *XhoI* (X), *EcoRI* (E), and *XmnI* (N) are as indicated. NCOs and COs are detected by digesting DNA with *EcoRI* and *XhoI* and then probing with *HIS4* sequences (blue bar, hisU). To detect JMs, genomic DNA is digested with *XmnI* and probed with *ARG4* sequences (black bar, argD). Probe sequences are described in Materials and Methods in the main text.
(TIF)

Figure S6 Identification of IS-JMs on 2D gels. (**A**) Analysis of JM formation via two-dimensional gel electrophoresis in *hop1Δ NDT80-IN* (CB2272) at the indicated time points. Gel conditions and species identification are as described for Figure 4. (**B**) The blot used in Figure 4D from *NDT80-IN smc6-56*, containing the 0 h and 7 h time points, was stripped and re-probed with the hisU probe, which only recognizes one homolog (P2) and detects the P2×P2 IS-JM (solid line) and the P1×P2 IH-JM (dashed line) as indicated in the schematic drawing. The grey square indicates the region that has been enlarged and enhanced to better visualize the JM spots in the lower panel. Species determined based on predicted size.
(TIF)

Figure S7 Independent experiments for two-dimensional analysis of JMs. JM analysis at indicated time points for *NDT80-IN SMC6* (CB2096) and *NDT80-IN smc6-56* (CB2097). Arrows above the blot images and below the graphs denote time of *NDT80* induction with β-estradiol (ED). Gel conditions are as described for Figure 4 and in Materials and Methods. Ratios of IS-JM and IH-JM species given under relevant images; quantifications of shown blots given to the right of each panel. Schematic diagram represents inferred JM species. (**A**) JM levels and species for cells undergoing meiosis under soft-shift conditions. (**B**) Cells were accumulated in an *ndt80* arrest at permissive temperature (green lines, −ED) until *NDT80* induction when cultures were shifted to non-permissive temperature (red lines, +ED) at 7 h. (**C**) Cells were accumulated in an *ndt80* arrest with soft-shift to non-permissive temperature (red lines, −ED). *NDT80* was induced concurrently with the shift to permissive temperature (green lines, +ED) at 7 h.
(TIF)

Figure S8 Smc6 protein levels. Western blots detecting an epitope-marked allele of *SMC6* (*SMC6-6HIS-3xFLAG*) in a wild-type background (CB1181) and a *rec8Δ* background (CB1430) by extracting protein and probing for anti-FLAG as described in Text S1 at the indicated time points. Anti-actin was used as a loading control.
(TIF)

Figure S9 Quantification methods for calculating the ratio of JM species and total JM levels from 2D gels. (**A**) Determination of the ratios of IS-JMs and IH-JMs. Image is from the 7 h time point from Figure 5D, *NDT80-IN smc6-56* (permissive to non-permissive). A single line was drawn to intersect the approximate centers of each JM spot using the Multi Gauge program (Fujifilm). The software generated a peak profile and the peaks representing each JM species were defined and selected after setting a threshold value as illustrated. As shown to the right, the software then generated intensity values, found from the area under each peak, and the subsequent ratios were calculated as signal/Σ JM signals. Because this method of analysis does not require using the parental bands as standards, images could be exposed for longer periods to get stronger signals in the JM region without being concerned about overexposure of the parental bands. It is also important to note that the sum of the two IS-JM signals was used for comparison. This is crucial because analyzing just one set of IS-JMs would be incorrect since the two homologs exhibit different levels of DSBs (Figure S5) [40]. Values in bold are those shown in Figure 5D. (**B**) Demonstration of how total JM levels were calculated from blot from the 7 h time point from Figure 6D, *NDT80-IN SMC6* (non-permissive to permissive). The areas corresponding to JM and parental regions (P1+P2) were selected using equal-sized regions of interest, plus an equivalent region near the JM region corresponding to background. Percent of Σ JMs was then determined by: (measured value for JM region−measured background value)/(measured value for JM region+measured value for parental region)×100.
(TIF)

Table S1 Yeast strains used in this study. All strains are derivatives of SK1 [91]. CB1017 was created by diploidizing K8379 (*MATa, ho::LYS2, ura3, leu2::hisG, trp1::hisG, his3::hisG, lys2*), a kind gift from the lab of Dr. Kim Nasmyth. Modifications to CB1017's genotype are indicated for each strain. Strains are MATa/α and homozygous for described loci unless otherwise indicated. The *rad50S* (*rad50-K181* = *rad50S*) strains were derived from NKY1002 [79] and were a gift from Dr. Kim Nasmyth's lab. Strains used for *HIS4LEU2* recombination assays were derived from NKY1303 (*MATa, ho::LYS2, lys2, leu2::hisG, ura3, arg4-Bgl2, his4B::LEU2-MluII*) and NKY1543 (*MATalpha, ho::LYS2, lys2, leu2::hisG, ura3, his4XLEU2-MluI::BamHI-URA3, arg4-Nsp*), which were originally generated in the lab of Dr. Nancy Kleckner and described in [12] and [77]. CenV-GFP and TelV-GFP strains were derived from FKY756 (*MATa, ho::LYS2, promURA3::tetR::GFP-LEU2, tetOx224-URA3*) and FKY4214 (*MATa/alpha, ho::LYS2, lys2, trp1, promURA3-TetR-GFP::LEU2, Bmh1::tetOx224-URA3, ura3, leu2::hisG, his3::hisG*), respectively. Strains containing *spo11::URA3 spo13::hisG* were derived from FKY1725 (*MATa, ho::LYS2, lys2, spo11::URA3, spo13::hisG, trp1::hisG, leu2, his3::hisG, ura3*). The strains used for JM and CO/NCO detection are descendants of MLS1827 (MATalpha, ho::LYS2, lys2, arg4del(*eco47III-hpa1), leu-R, ura3, his4del(Sal1-Cla1)::URA3-del(Sma1-Eco47III)-arg4-EcPal(1691)*) and MLS1076 (*MATa, ho::LYS2, lys2, arg4del(eco47III-hpaI), cyh2-z, ura3, leu2-RV::URA3-(Sma1-Eco47III)-[ARG4* cloned]), which were created in the lab of Dr. Michael Lichten and originally described in [11]. Strains harbouring the *NDT80-IN* allele were derived from FKY4453 (*MATa/alpha, ho::LYS2, lys2, ura3, leu2::hisG, trp1::hisG, his3::hisG, pGAL-NDT80::TRP1, ura3::pGPD1-GAL4(848).ER::URA3*). The NKY, FKY and MLS strains are kind gifts from the lab of Dr. Franz Klein.
(PDF)

Text S1 Supplementary methods and references.
(DOCX)

## References

[1] Kleckner N (1996) Meiosis: how could it work? Proc Natl Acad Sci U S A 93: 8167–8174. 8710842 10.1073/pnas.93.16.8167 PMC38641

[2] Keeney S (2008) Spo11 and the Formation of DNA Double-Strand Breaks in Meiosis. Genome Dyn Stab 2: 81–123. 21927624 10.1007/7050_2007_026 PMC3172816

[3] Keeney S, Giroux CN, Kleckner N (1997) Meiosis-specific DNA double-strand breaks are catalyzed by Spo11, a member of a widely conserved protein family. Cell 88: 375–384. 9039264 10.1016/s0092-8674(00)81876-0

[4] Mimitou EP, Symington LS (2008) Sae2, Exo1 and Sgs1 collaborate in DNA double-strand break processing. Nature 455: 770–774. 18806779 10.1038/nature07312 PMC3818707

[5] Schwartz EK, Heyer WD (2011) Processing of joint molecule intermediates by structure-selective endonucleases during homologous recombination in eukaryotes. Chromosoma 120: 109–127. 21369956 10.1007/s00412-010-0304-7 PMC3057012

[6] Hunter N, Kleckner N (2001) The single-end invasion: an asymmetric intermediate at the double-strand break to double-holliday junction transition of meiotic recombination. Cell 106: 59–70. 11461702 10.1016/s0092-8674(01)00430-5

[7] Paques F, Haber JE (1999) Multiple pathways of recombination induced by double-strand breaks in *Saccharomyces cerevisiae* . Microbiol Mol Biol Rev 63: 349–404. 10357855 10.1128/mmbr.63.2.349-404.1999 PMC98970

[8] De Muyt A, Jessop L, Kolar E, Sourirajan A, Chen J, et al (2012) BLM helicase ortholog Sgs1 is a central regulator of meiotic recombination intermediate metabolism. Mol Cell 46: 43–53. 22500736 10.1016/j.molcel.2012.02.020 PMC3328772

[9] Schwacha A, Kleckner N (1995) Identification of double Holliday junctions as intermediates in meiotic recombination. Cell 83: 783–791. 8521495 10.1016/0092-8674(95)90191-4

[10] Youds JL, Boulton SJ (2011) The choice in meiosis - defining the factors that influence crossover or non-crossover formation. J Cell Sci 124: 501–513. 21282472 10.1242/jcs.074427

[11] Allers T, Lichten M (2001) Differential timing and control of noncrossover and crossover recombination during meiosis. Cell 106: 47–57. 11461701 10.1016/s0092-8674(01)00416-0

[12] Storlazzi A, Xu L, Cao L, Kleckner N (1995) Crossover and noncrossover recombination during meiosis: timing and pathway relationships. Proc Natl Acad Sci U S A 92: 8512–8516. 7667321 10.1073/pnas.92.18.8512 PMC41187

[13] Borner GV, Kleckner N, Hunter N (2004) Crossover/noncrossover differentiation, synaptonemal complex formation, and regulatory surveillance at the leptotene/zygotene transition of meiosis. Cell 117: 29–45. 15066280 10.1016/s0092-8674(04)00292-2

[14] Lynn A, Soucek R, Borner GV (2007) ZMM proteins during meiosis: crossover artists at work. Chromosome Res 15: 591–605. 17674148 10.1007/s10577-007-1150-1

[15] Shinohara M, Oh SD, Hunter N, Shinohara A (2008) Crossover assurance and crossover interference are distinctly regulated by the ZMM proteins during yeast meiosis. Nat Genet 40: 299–309. 18297071 10.1038/ng.83

[16] Falk JE, Chan AC, Hoffmann E, Hochwagen A (2010) A Mec1- and PP4-dependent checkpoint couples centromere pairing to meiotic recombination. Dev Cell 19: 599–611. 20951350 10.1016/j.devcel.2010.09.006

[17] Zakharyevich K, Tang S, Ma Y, Hunter N (2012) Delineation of joint molecule resolution pathways in meiosis identifies a crossover-specific resolvase. Cell 149: 334–347. 22500800 10.1016/j.cell.2012.03.023 PMC3377385

[18] de los Santos T, Hunter N, Lee C, Larkin B, Loidl J, et al (2003) The Mus81/Mms4 endonuclease acts independently of double-Holliday junction resolution to promote a distinct subset of crossovers during meiosis in budding yeast. Genetics 164: 81–94. 12750322 10.1093/genetics/164.1.81 PMC1462551

[19] Holloway JK, Booth J, Edelmann W, McGowan CH, Cohen PE (2008) *MUS81* generates a subset of *MLH1–MLH3*-independent crossovers in mammalian meiosis. PLoS Genet 4: e1000186. 18787696 10.1371/journal.pgen.1000186 PMC2525838

[20] Osman F, Dixon J, Doe CL, Whitby MC (2003) Generating crossovers by resolution of nicked Holliday junctions: a role for Mus81-Eme1 in meiosis. Mol Cell 12: 761–774. 14527420 10.1016/s1097-2765(03)00343-5

[21] Smith GR, Boddy MN, Shanahan P, Russell P (2003) Fission yeast Mus81.Eme1 Holliday junction resolvase is required for meiotic crossing over but not for gene conversion. Genetics 165: 2289–2293. 14704204 10.1093/genetics/165.4.2289 PMC1462924

[22] Cromie G, Smith GR (2008) Meiotic Recombination in *Schizosaccharomyces pombe*: A Paradigm for Genetic and Molecular Analysis. Genome Dyn Stab 3: 195. 20157622 10.1007/7050_2007_025 PMC2820269

[23] Cromie GA, Hyppa RW, Smith GR (2008) The fission yeast BLM homolog Rqh1 promotes meiotic recombination. Genetics 179: 1157–1167. 18562672 10.1534/genetics.108.088955 PMC2475723

[24] Cromie GA, Hyppa RW, Taylor AF, Zakharyevich K, Hunter N, et al (2006) Single Holliday junctions are intermediates of meiotic recombination. Cell 127: 1167–1178. 17174892 10.1016/j.cell.2006.09.050 PMC2803030

[25] Filipski J, Mucha M (2002) Structure, function and DNA composition of *Saccharomyces cerevisiae* chromatin loops. Gene 300: 63–68. 12468087 10.1016/s0378-1119(02)00848-x

[26] Blat Y, Protacio RU, Hunter N, Kleckner N (2002) Physical and functional interactions among basic chromosome organizational features govern early steps of meiotic chiasma formation. Cell 111: 791–802. 12526806 10.1016/s0092-8674(02)01167-4

[27] Kleckner N (2006) Chiasma formation: chromatin/axis interplay and the role(s) of the synaptonemal complex. Chromosoma 115: 175–194. 16555016 10.1007/s00412-006-0055-7

[28] Acquaviva L, Szekvolgyi L, Dichtl B, Dichtl BS, de La Roche Saint Andre C, et al (2013) The COMPASS subunit Spp1 links histone methylation to initiation of meiotic recombination. Science 339: 215–218. 23160953 10.1126/science.1225739

[29] Sommermeyer V, Beneut C, Chaplais E, Serrentino ME, Borde V (2013) Spp1, a member of the Set1 Complex, promotes meiotic DSB formation in promoters by tethering histone H3K4 methylation sites to chromosome axes. Mol Cell 49: 43–54. 23246437 10.1016/j.molcel.2012.11.008

[30] Panizza S, Mendoza MA, Berlinger M, Huang L, Nicolas A, et al (2011) Spo11-accessory proteins link double-strand break sites to the chromosome axis in early meiotic recombination. Cell 146: 372–383. 21816273 10.1016/j.cell.2011.07.003

[31] Storlazzi A, Gargano S, Ruprich-Robert G, Falque M, David M, et al (2010) Recombination proteins mediate meiotic spatial chromosome organization and pairing. Cell 141: 94–106. 20371348 10.1016/j.cell.2010.02.041 PMC2851631

[32] Schwacha A, Kleckner N (1997) Interhomolog bias during meiotic recombination: meiotic functions promote a highly differentiated interhomolog-only pathway. Cell 90: 1123–1135. 9323140 10.1016/s0092-8674(00)80378-5

[33] Bishop DK, Park D, Xu L, Kleckner N (1992) *DMC1*: a meiosis-specific yeast homolog of E. coli recA required for recombination, synaptonemal complex formation, and cell cycle progression. Cell 69: 439–456. 1581960 10.1016/0092-8674(92)90446-j

[34] Shinohara A, Gasior S, Ogawa T, Kleckner N, Bishop DK (1997) *Saccharomyces cerevisiae* recA homologues *RAD51* and *DMC1* have both distinct and overlapping roles in meiotic recombination. Genes Cells 2: 615–629. 9427283 10.1046/j.1365-2443.1997.1480347.x

[35] Niu H, Wan L, Baumgartner B, Schaefer D, Loidl J, et al (2005) Partner choice during meiosis is regulated by Hop1-promoted dimerization of Mek1. Mol Biol Cell 16: 5804–5818. 16221890 10.1091/mbc.E05-05-0465 PMC1289423

[36] Hollingsworth NM, Ponte L (1997) Genetic interactions between *HOP1*, *RED1* and *MEK1* suggest that *MEK1* regulates assembly of axial element components during meiosis in the yeast *Saccharomyces cerevisiae* . Genetics 147: 33–42. 9286666 10.1093/genetics/147.1.33 PMC1208117

[37] Carballo JA, Johnson AL, Sedgwick SG, Cha RS (2008) Phosphorylation of the axial element protein Hop1 by Mec1/Tel1 ensures meiotic interhomolog recombination. Cell 132: 758–770. 18329363 10.1016/j.cell.2008.01.035

[38] Revenkova E, Jessberger R (2006) Shaping meiotic prophase chromosomes: cohesins and synaptonemal complex proteins. Chromosoma 115: 235–240. 16518630 10.1007/s00412-006-0060-x

[39] Kugou K, Fukuda T, Yamada S, Ito M, Sasanuma H, et al (2009) Rec8 guides canonical Spo11 distribution along yeast meiotic chromosomes. Mol Biol Cell 20: 3064–3076. 19439448 10.1091/mbc.E08-12-1223 PMC2704158

[40] Kim KP, Weiner BM, Zhang L, Jordan A, Dekker J, et al (2010) Sister cohesion and structural axis components mediate homolog bias of meiotic recombination. Cell 143: 924–937. 21145459 10.1016/j.cell.2010.11.015 PMC3033573

[41] Goldfarb T, Lichten M (2010) Frequent and efficient use of the sister chromatid for DNA double-strand break repair during budding yeast meiosis. PLoS Biol 8: e1000520. 20976044 10.1371/journal.pbio.1000520 PMC2957403

[42] Schwacha A, Kleckner N (1994) Identification of joint molecules that form frequently between homologs but rarely between sister chromatids during yeast meiosis. Cell 76: 51–63. 8287479 10.1016/0092-8674(94)90172-4

[43] Peters JM, Nishiyama T (2012) Sister chromatid cohesion. Cold Spring Harb Perspect Biol 4: a011130. 23043155 10.1101/cshperspect.a011130 PMC3536341

[44] Fousteri MI, Lehmann AR (2000) A novel SMC protein complex in *Schizosaccharomyces pombe* contains the Rad18 DNA repair protein. EMBO J 19: 1691–1702. 10747036 10.1093/emboj/19.7.1691 PMC310237

[45] Andrews EA, Palecek J, Sergeant J, Taylor E, Lehmann AR, et al (2005) Nse2, a component of the Smc5-6 complex, is a SUMO ligase required for the response to DNA damage. Mol Cell Biol 25: 185–196. 15601841 10.1128/MCB.25.1.185-196.2005 PMC538766

[46] Lehmann AR, Walicka M, Griffiths DJ, Murray JM, Watts FZ, et al (1995) The *rad18* gene of *Schizosaccharomyces pombe* defines a new subgroup of the SMC superfamily involved in DNA repair. Mol Cell Biol 15: 7067–7080. 8524274 10.1128/mcb.15.12.7067 PMC230962

[47] Kegel A, Betts-Lindroos H, Kanno T, Jeppsson K, Strom L, et al (2011) Chromosome length influences replication-induced topological stress. Nature 471: 392–396. 21368764 10.1038/nature09791

[48] Strom L, Lindroos HB, Shirahige K, Sjogren C (2004) Postreplicative recruitment of cohesin to double-strand breaks is required for DNA repair. Mol Cell 16: 1003–1015. 15610742 10.1016/j.molcel.2004.11.026

[49] Lindroos HB, Strom L, Itoh T, Katou Y, Shirahige K, et al (2006) Chromosomal association of the Smc5/6 complex reveals that it functions in differently regulated pathways. Mol Cell 22: 755–767. 16793545 10.1016/j.molcel.2006.05.014

[50] De Piccoli G, Cortes-Ledesma F, Ira G, Torres-Rosell J, Uhle S, et al (2006) Smc5-Smc6 mediate DNA double-strand-break repair by promoting sister-chromatid recombination. Nat Cell Biol 8: 1032–1034. 16892052 10.1038/ncb1466 PMC4493748

[51] Branzei D, Sollier J, Liberi G, Zhao X, Maeda D, et al (2006) Ubc9- and mms21-mediated sumoylation counteracts recombinogenic events at damaged replication forks. Cell 127: 509–522. 17081974 10.1016/j.cell.2006.08.050

[52] Sollier J, Driscoll R, Castellucci F, Foiani M, Jackson SP, et al (2009) The *Saccharomyces cerevisiae* Esc2 and Smc5-6 proteins promote sister chromatid junction-mediated intra-S repair. Mol Biol Cell 20: 1671–1682. 19158389 10.1091/mbc.E08-08-0875 PMC2655255

[53] Chavez A, George V, Agrawal V, Johnson FB (2010) Sumoylation and the structural maintenance of chromosomes (Smc) 5/6 complex slow senescence through recombination intermediate resolution. J Biol Chem 285: 11922–11930. 20159973 10.1074/jbc.M109.041277 PMC2852929

[54] Morikawa H, Morishita T, Kawane S, Iwasaki H, Carr AM, et al (2004) Rad62 protein functionally and physically associates with the smc5/smc6 protein complex and is required for chromosome integrity and recombination repair in fission yeast. Mol Cell Biol 24: 9401–9413. 15485909 10.1128/MCB.24.21.9401-9413.2004 PMC522231

[55] Hwang JY, Smith S, Ceschia A, Torres-Rosell J, Aragon L, et al (2008) Smc5-Smc6 complex suppresses gross chromosomal rearrangements mediated by break-induced replications. DNA Repair (Amst) 7: 1426–1436. 18585101 10.1016/j.dnarep.2008.05.006 PMC2585499

[56] Chen YH, Choi K, Szakal B, Arenz J, Duan X, et al (2009) Interplay between the Smc5/6 complex and the Mph1 helicase in recombinational repair. Proc Natl Acad Sci U S A 106: 21252–21257. 19995966 10.1073/pnas.0908258106 PMC2795505

[57] Bickel JS, Chen L, Hayward J, Yeap SL, Alkers AE, et al (2010) Structural maintenance of chromosomes (SMC) proteins promote homolog-independent recombination repair in meiosis crucial for germ cell genomic stability. PLoS Genet 6: e1001028. 20661436 10.1371/journal.pgen.1001028 PMC2908675

[58] Pebernard S, McDonald WH, Pavlova Y, Yates JR 3rd, Boddy MN (2004) Nse1, Nse2, and a novel subunit of the Smc5-Smc6 complex, Nse3, play a crucial role in meiosis. Mol Biol Cell 15: 4866–4876. 15331764 10.1091/mbc.E04-05-0436 PMC524734

[59] Wehrkamp-Richter S, Hyppa RW, Prudden J, Smith GR, Boddy MN (2012) Meiotic DNA joint molecule resolution depends on Nse5-Nse6 of the Smc5-Smc6 holocomplex. Nucleic Acids Res 40: 9633–9646. 22855558 10.1093/nar/gks713 PMC3479181

[60] Farmer S, San-Segundo PA, Aragon L (2011) The Smc5-Smc6 complex is required to remove chromosome junctions in meiosis. PLoS One 6: e20948. 21731634 10.1371/journal.pone.0020948 PMC3120815

[61] Onoda F, Takeda M, Seki M, Maeda D, Tajima J, et al (2004) *SMC6* is required for MMS-induced interchromosomal and sister chromatid recombinations in *Saccharomyces cerevisiae* . DNA Repair (Amst) 3: 429–439. 15010319 10.1016/j.dnarep.2003.12.007

[62] Lee BH, Amon A (2003) Role of Polo-like kinase *CDC5* in programming meiosis I chromosome segregation. Science 300: 482–486. 12663816 10.1126/science.1081846

[63] Grandin N, Reed SI (1993) Differential function and expression of *Saccharomyces cerevisiae* B-type cyclins in mitosis and meiosis. Mol Cell Biol 13: 2113–2125. 8455600 10.1128/mcb.13.4.2113-2125.1993 PMC359532

[64] Dahmann C, Futcher B (1995) Specialization of B-type cyclins for mitosis or meiosis in *S. cerevisiae* . Genetics 140: 957–963. 7672594 10.1093/genetics/140.3.957 PMC1206679

[65] Neiman AM (2011) Sporulation in the budding yeast *Saccharomyces cerevisiae* . Genetics 189: 737–765. 22084423 10.1534/genetics.111.127126 PMC3213374

[66] Sym M, Engebrecht JA, Roeder GS (1993) *ZIP1* is a synaptonemal complex protein required for meiotic chromosome synapsis. Cell 72: 365–378. 7916652 10.1016/0092-8674(93)90114-6

[67] Roeder GS, Bailis JM (2000) The pachytene checkpoint. Trends Genet 16: 395–403. 10973068 10.1016/s0168-9525(00)02080-1

[68] Tung KS, Hong EJ, Roeder GS (2000) The pachytene checkpoint prevents accumulation and phosphorylation of the meiosis-specific transcription factor Ndt80. Proc Natl Acad Sci U S A 97: 12187–12192. 11035815 10.1073/pnas.220464597 PMC17316

[69] Chu S, Herskowitz I (1998) Gametogenesis in yeast is regulated by a transcriptional cascade dependent on Ndt80. Mol Cell 1: 685–696. 9660952 10.1016/s1097-2765(00)80068-4

[70] Xu L, Ajimura M, Padmore R, Klein C, Kleckner N (1995) *NDT80*, a meiosis-specific gene required for exit from pachytene in *Saccharomyces cerevisiae* . Mol Cell Biol 15: 6572–6581. 8524222 10.1128/mcb.15.12.6572 PMC230910

[71] Katis VL, Matos J, Mori S, Shirahige K, Zachariae W, et al (2004) Spo13 facilitates monopolin recruitment to kinetochores and regulates maintenance of centromeric cohesion during yeast meiosis. Curr Biol 14: 2183–2196. 15620645 10.1016/j.cub.2004.12.020

[72] Klapholz S, Esposito RE (1980) Isolation of *SPO12-1* and *SPO13-1* from a natural variant of yeast that undergoes a single meiotic division. Genetics 96: 567–588. 7021311 10.1093/genetics/96.3.567 PMC1214362

[73] Michaelis C, Ciosk R, Nasmyth K (1997) Cohesins: chromosomal proteins that prevent premature separation of sister chromatids. Cell 91: 35–45. 9335333 10.1016/s0092-8674(01)80007-6

[74] Tanaka T, Cosma MP, Wirth K, Nasmyth K (1999) Identification of cohesin association sites at centromeres and along chromosome arms. Cell 98: 847–858. 10499801 10.1016/s0092-8674(00)81518-4

[75] Klein F, Mahr P, Galova M, Buonomo SB, Michaelis C, et al (1999) A central role for cohesins in sister chromatid cohesion, formation of axial elements, and recombination during yeast meiosis. Cell 98: 91–103. 10412984 10.1016/S0092-8674(00)80609-1

[76] Buonomo SB, Clyne RK, Fuchs J, Loidl J, Uhlmann F, et al (2000) Disjunction of homologous chromosomes in meiosis I depends on proteolytic cleavage of the meiotic cohesin Rec8 by separin. Cell 103: 387–398. 11081626 10.1016/s0092-8674(00)00131-8

[77] Xu L, Kleckner N (1995) Sequence non-specific double-strand breaks and interhomolog interactions prior to double-strand break formation at a meiotic recombination hot spot in yeast. EMBO J 14: 5115–5128. 7588640 10.1002/j.1460-2075.1995.tb00194.x PMC394615

[78] Cao L, Alani E, Kleckner N (1990) A pathway for generation and processing of double-strand breaks during meiotic recombination in *S. cerevisiae* . Cell 61: 1089–1101. 2190690 10.1016/0092-8674(90)90072-m

[79] Alani E, Padmore R, Kleckner N (1990) Analysis of wild-type and *rad50* mutants of yeast suggests an intimate relationship between meiotic chromosome synapsis and recombination. Cell 61: 419–436. 2185891 10.1016/0092-8674(90)90524-i

[80] Jessop L, Lichten M (2008) Mus81/Mms4 endonuclease and Sgs1 helicase collaborate to ensure proper recombination intermediate metabolism during meiosis. Mol Cell 31: 313–323. 18691964 10.1016/j.molcel.2008.05.021 PMC2584117

[81] Oh SD, Lao JP, Taylor AF, Smith GR, Hunter N (2008) RecQ helicase, Sgs1, and XPF family endonuclease, Mus81-Mms4, resolve aberrant joint molecules during meiotic recombination. Mol Cell 31: 324–336. 18691965 10.1016/j.molcel.2008.07.006 PMC2587322

[82] Carlile TM, Amon A (2008) Meiosis I is established through division-specific translational control of a cyclin. Cell 133: 280–291. 18423199 10.1016/j.cell.2008.02.032 PMC2396536

[83] Benjamin KR, Zhang C, Shokat KM, Herskowitz I (2003) Control of landmark events in meiosis by the CDK Cdc28 and the meiosis-specific kinase Ime2. Genes Dev 17: 1524–1539. 12783856 10.1101/gad.1101503 PMC196082

[84] Sourirajan A, Lichten M (2008) Polo-like kinase Cdc5 drives exit from pachytene during budding yeast meiosis. Genes Dev 22: 2627–2632. 18832066 10.1101/gad.1711408 PMC2559907

[85] Allers T, Lichten M (2001) Intermediates of yeast meiotic recombination contain heteroduplex DNA. Mol Cell 8: 225–231. 11511375 10.1016/s1097-2765(01)00280-5

[86] Jessop L, Allers T, Lichten M (2005) Infrequent co-conversion of markers flanking a meiotic recombination initiation site in *Saccharomyces cerevisiae* . Genetics 169: 1353–1367. 15654098 10.1534/genetics.104.036509 PMC1449552

[87] Bell L, Byers B (1983) Separation of Branched from Linear DNA by Two-Dimensional Gel-Electrophoresis. Analytical Biochemistry 130: 527–535. 6869840 10.1016/0003-2697(83)90628-0

[88] Hollingsworth NM, Goetsch L, Byers B (1990) The *HOP1* gene encodes a meiosis-specific component of yeast chromosomes. Cell 61: 73–84. 2107981 10.1016/0092-8674(90)90216-2

[89] Bermudez-Lopez M, Ceschia A, de Piccoli G, Colomina N, Pasero P, et al (2010) The Smc5/6 complex is required for dissolution of DNA-mediated sister chromatid linkages. Nucleic Acids Res 38: 6502–6512. 20571088 10.1093/nar/gkq546 PMC2965248

[90] Blitzblau HG, Bell GW, Rodriguez J, Bell SP, Hochwagen A (2007) Mapping of meiotic single-stranded DNA reveals double-stranded-break hotspots near centromeres and telomeres. Curr Biol 17: 2003–2012. 18060788 10.1016/j.cub.2007.10.066

[91] Kane SM, Roth R (1974) Carbohydrate metabolism during ascospore development in yeast. J Bacteriol 118: 8–14. 4595206 10.1128/jb.118.1.8-14.1974 PMC246633

[92] Longtine MS, McKenzie A 3rd, Demarini DJ, Shah NG, Wach A, et al (1998) Additional modules for versatile and economical PCR-based gene deletion and modification in *Saccharomyces cerevisiae* . Yeast 14: 953–961. 9717241 10.1002/(SICI)1097-0061(199807)14:10<953::AID-YEA293>3.0.CO;2-U

[93] Elrod SL, Chen SM, Schwartz K, Shuster EO (2009) Optimizing sporulation conditions for different *Saccharomyces cerevisiae* strain backgrounds. Methods Mol Biol 557: 21–26. 19799173 10.1007/978-1-59745-527-5_2

[94] Oh SD, Jessop L, Lao JP, Allers T, Lichten M, et al (2009) Stabilization and electrophoretic analysis of meiotic recombination intermediates in *Saccharomyces cerevisiae* . Methods Mol Biol 557: 209–234. 19799185 10.1007/978-1-59745-527-5_14

[95] Allers T, Lichten M (2000) A method for preparing genomic DNA that restrains branch migration of Holliday junctions. Nucleic Acids Res 28: e6. 10606674 10.1093/nar/28.2.e6 PMC102538

[96] Nairz K, Klein F (1997) *mre11S*–a yeast mutation that blocks double-strand-break processing and permits nonhomologous synapsis in meiosis. Genes Dev 11: 2272–2290. 9303542 10.1101/gad.11.17.2272 PMC275393

