# Supplementary figures and images for: Inhibition of the Smc5/6 Complex during Meiosis Perturbs Joint Molecule Formation and Resolution without Significantly Changing Crossover or Non-crossover Levels
Source: PLoS Genet. 2013 Nov 7;9(11):e1003898. doi: 10.1371/journal.pgen.1003898 (PMC3820751; doi:10.1371/journal.pgen.1003898)

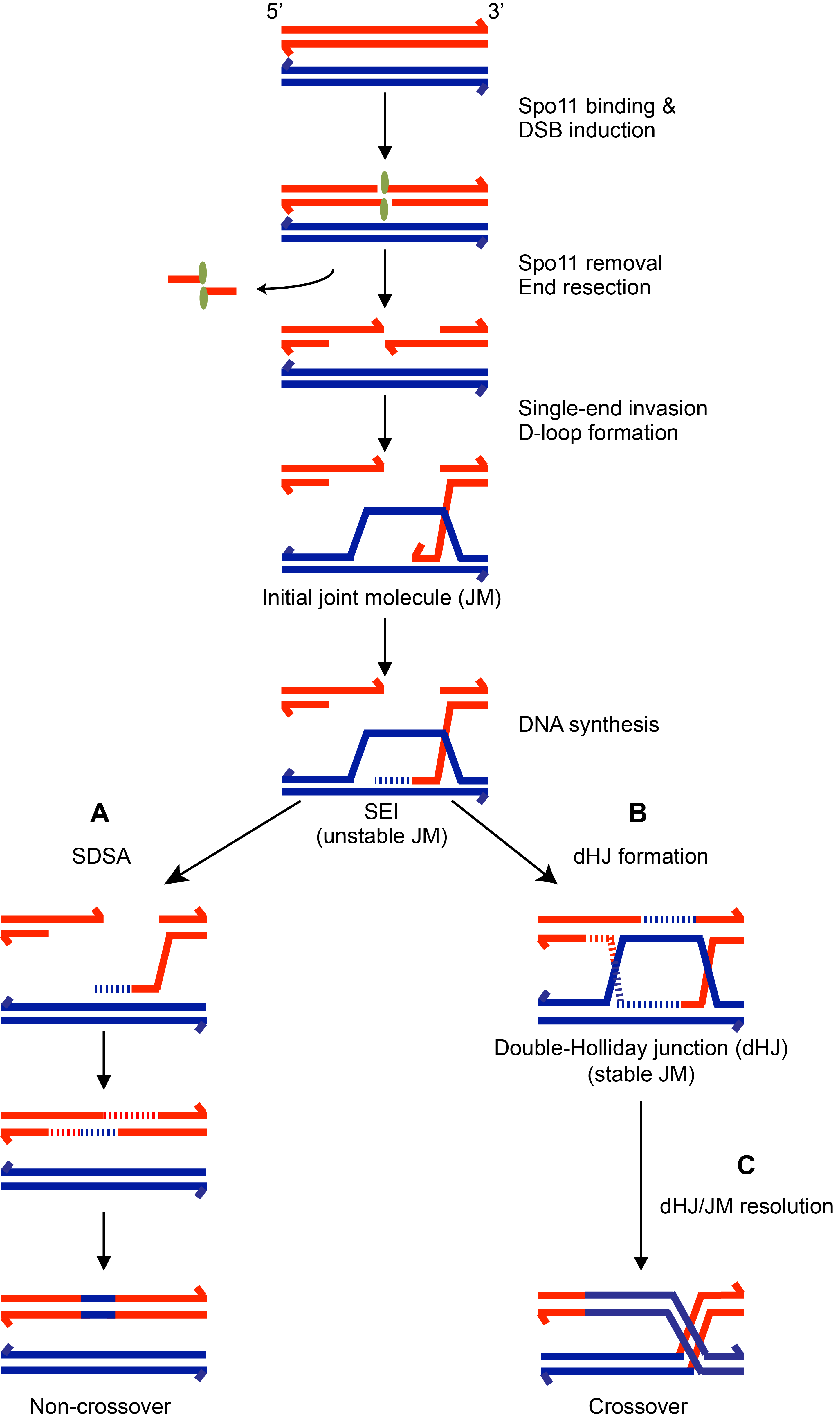

Supplement: Figure S1 — Schematic representation of meiotic recombination. Meiotic recombination is initiated by Spo11-catalyzed DNA double-strand breaks (DSBs). Spo11 is removed from the DNA in the form of Spo11-oligonucleotide complexes, allowing the 5′ ends of the DSB to be resected to generate 3′ single-stranded overhangs coated by Rad51 and Dmc1 (not shown) that can invade a homologous strand for repair. Strand invasion gives rise to a D-loop, forming an initial joint molecule (JM) intermediate. Following stabilization and DNA synthesis, the initial JM gives rise to another transient JM species called the single-end invasion (SEI). (A) The SEI can be quickly dissociated to re-ligate the newly synthesized DNA end to the complementary free break end in a process called synthesis-dependent strand annealing (SDSA). Additional DNA synthesis and ligation yields a mature non-crossover product. (B) Alternatively, the SEI can be stabilized to facilitate capture of the second 3′ DSB end via engagement of the intact homologous strand. Further processing yields gives rise to a stable JM intermediate known as a double-Holliday junction (dHJ). (C) Endonuclease-mediated resolution of the dHJ yields primarily crossover products. (TIF) [file pgen.1003898.s001.tif]

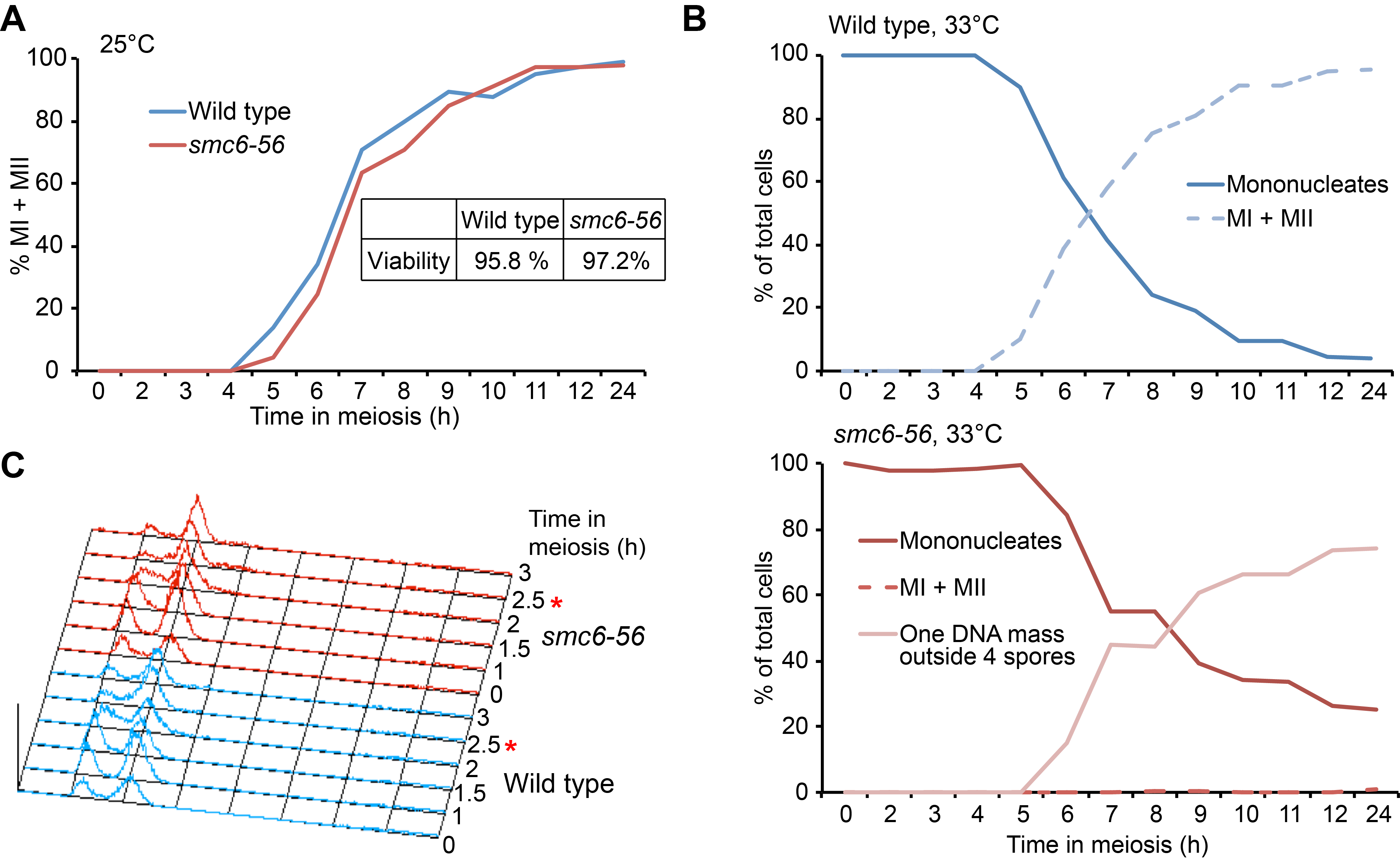

Supplement: Figure S2 — Segregation, viability and FACS profiles from wild-type and smc6-56 strains. (A) Meiotic progression and viability of wild type (CB1017) and smc6-56 (CB1032) at permissive temperature (25°C). Progression indicated as percent of MI+MII cells observed via DAPI staining of fixed whole cells, N = 200. Spore viability determined after dissection of 72 spores following sporulation for 3 days at permissive temperature. (B) Meiotic progression in wild type (blue curves, top) and smc6-56 (red curves, bottom) at non-permissive temperature (33°C). The smc6-56 mutant does not divide its nuclei and instead forms cells containing one DNA mass outside of four spores as described in Figure 1 but also has a population of cells that remain mononucleate when kept at non-permissive temperature from the time of transfer into meiotic media. N = 200. (C) FACS profiles from wild type (blue) and smc6-56 (red) undergoing meiosis under soft-shift conditions. Cultures were kept at permissive temperature (25°C) until 2.5 hours after meiotic induction (red asterisk) when the majority of the cells in the wild type and mutant had completed replication and the temperature was raised to non-permissive (33°C). (TIF) [file pgen.1003898.s002.tif]

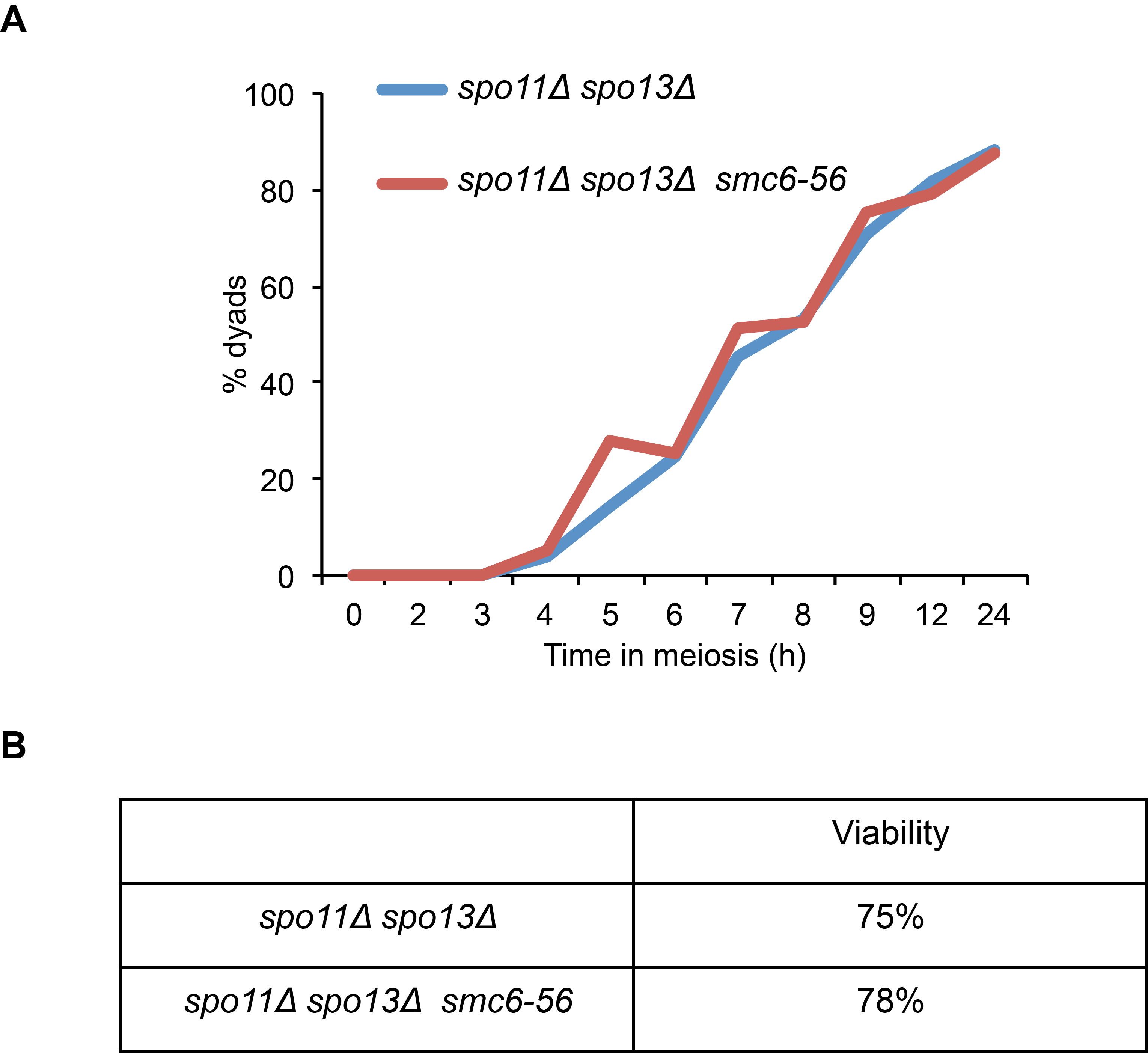

Supplement: Figure S3 — Segregation and spore viability in a spo11Δ spo13Δ background. (A) Percent dyad formation in spo11Δ spo13Δ (CB1466) and smc6-56 spo11Δ spo13Δ (CB1465) undergoing meiosis under soft-shift conditions. N = 200. (B) Spore viability after dissection of 36 spores for spo11Δ spo13Δ (CB1466) and smc6-56 spo11Δ spo13Δ (CB1465) after sporulation for three days at non-permissive temperature. Spores were grown for 3 days at permissive temperature. (TIF) [file pgen.1003898.s003.tif]

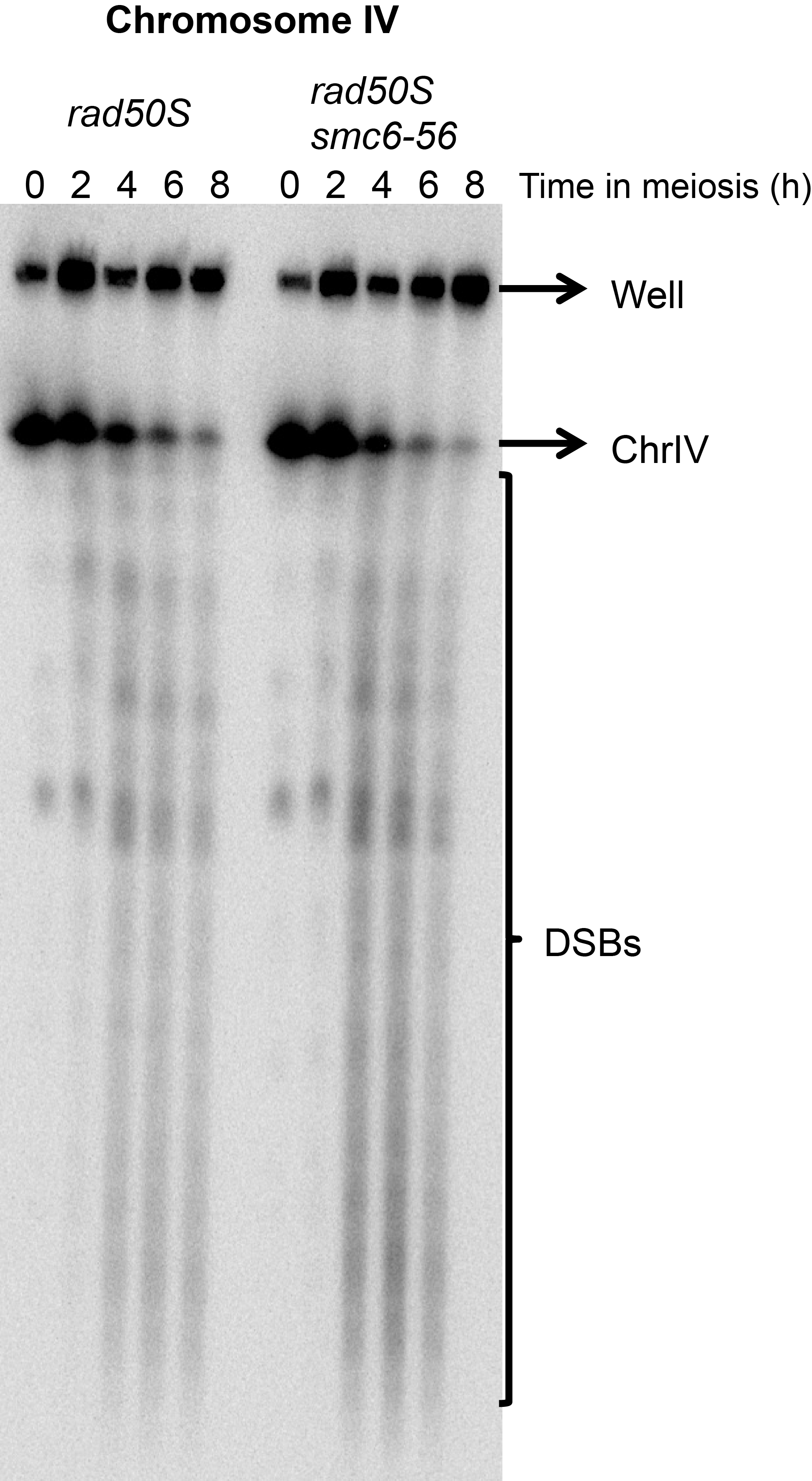

Supplement: Figure S4 — Whole-chromosome break pattern of chromosome IV in a rad50S background. Analysis of whole-chromosome break pattern for chromosome IV for rad50S (CB58) and rad50S smc6-56 (CB1360) strains undergoing meiosis under soft-shift conditions. At the indicated time points, cells were isolated and treated for DNA extraction and subsequent pulse-field gel electrophoresis as described in Text S1. (TIF) [file pgen.1003898.s004.tif]

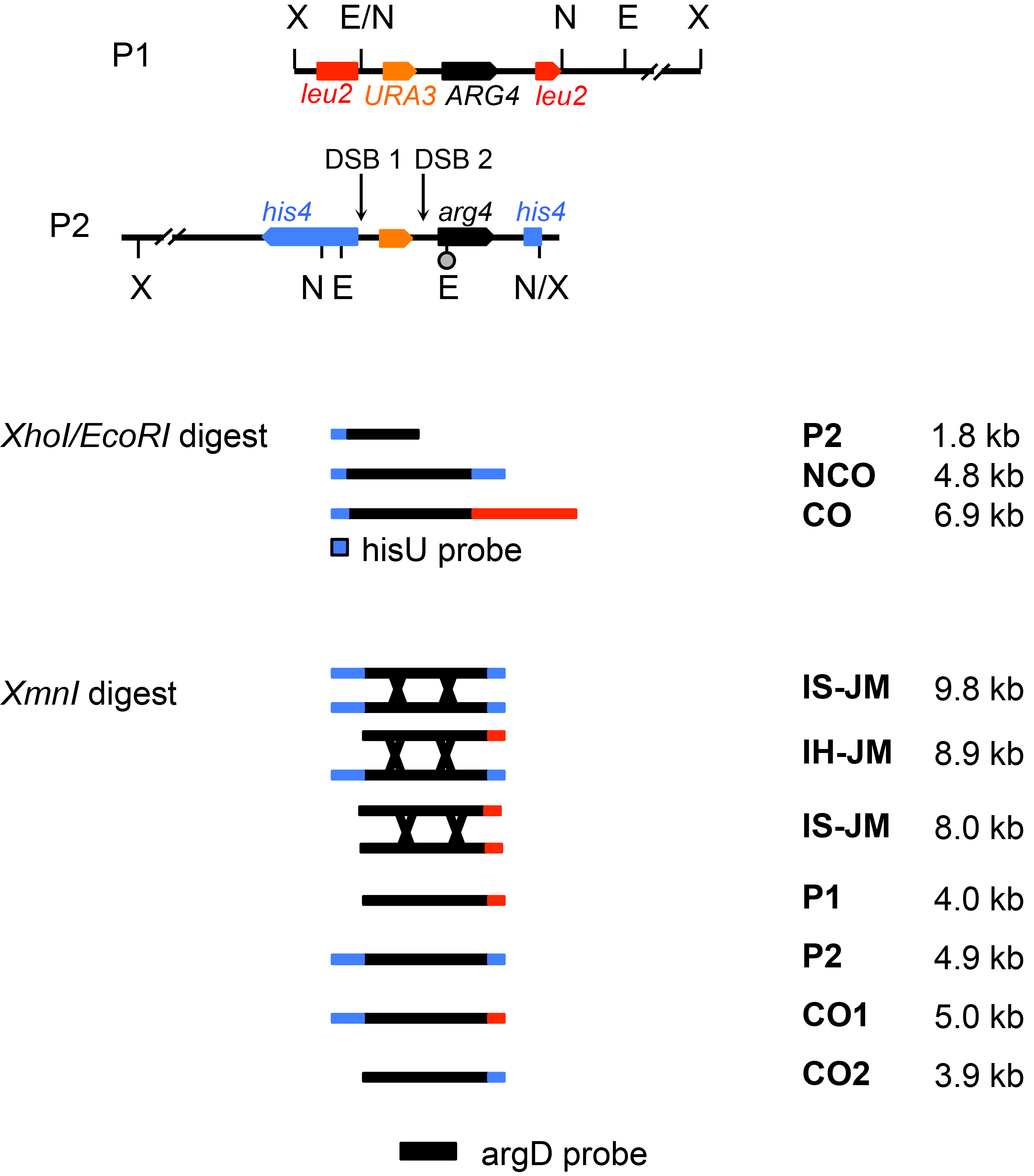

Supplement: Figure S5 — The URA3-ARG4 recombination hotspot on chromosome III. Figure is adapted from that shown in [84]. The URA3-ARG4 construct is inserted at LEU2 on one homolog (P1) and at HIS4 on the other homolog (P2). P2 contains an EcoRI site-containing palindrome (indicated by the grey circle) in the ARG4 sequence, denoted arg4-EcPal. The restriction sites XhoI (X), EcoRI (E), and XmnI (N) are as indicated. NCOs and COs are detected by digesting DNA with EcoRI and XhoI and then probing with HIS4 sequences (blue bar, hisU). To detect JMs, genomic DNA is digested with XmnI and probed with ARG4 sequences (black bar, argD). Probe sequences are described in Materials and Methods in the main text. (TIF) [file pgen.1003898.s005.tif]

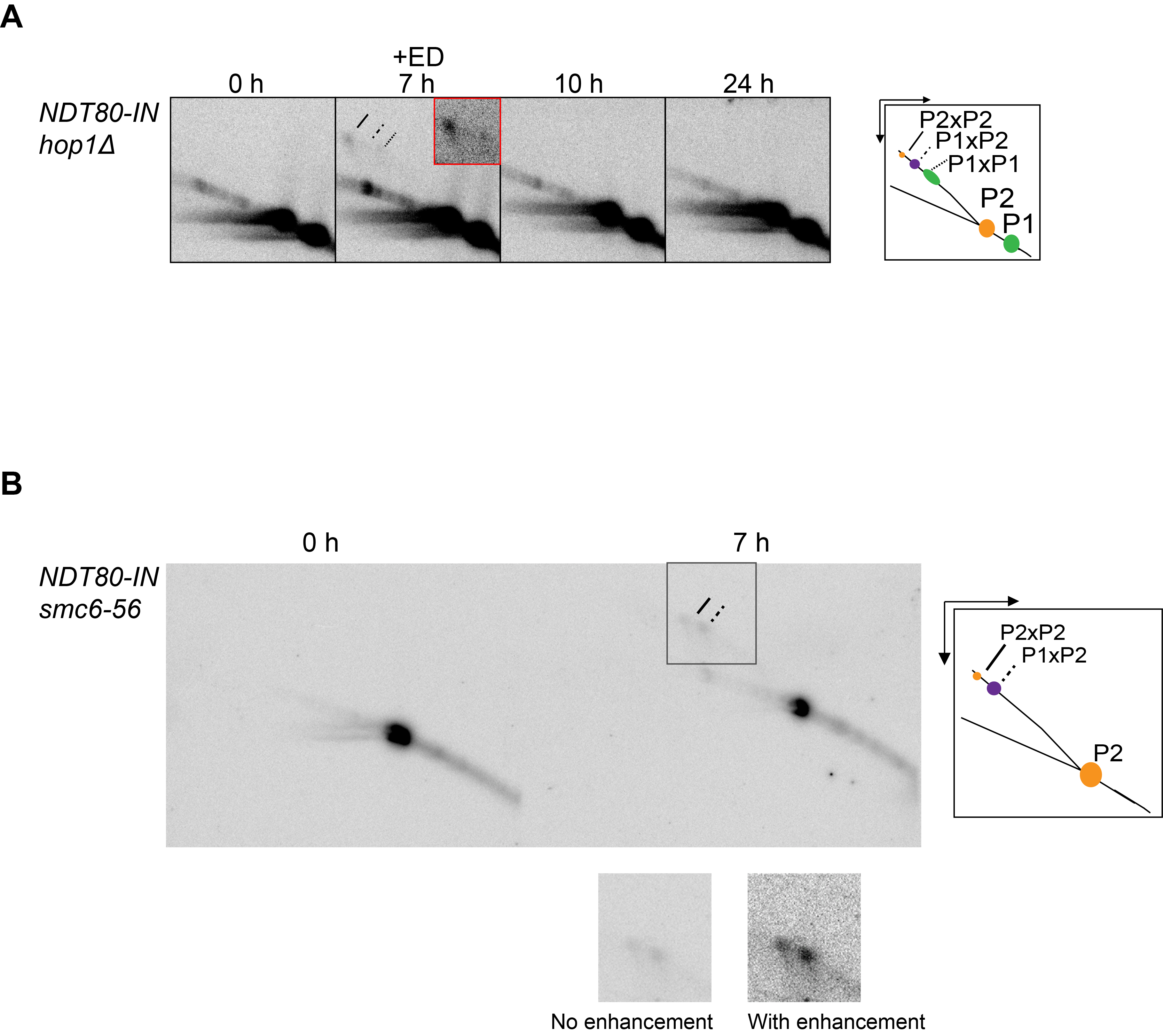

Supplement: Figure S6 — Identification of IS-JMs on 2D gels. (A) Analysis of JM formation via two-dimensional gel electrophoresis in hop1Δ NDT80-IN (CB2272) at the indicated time points. Gel conditions and species identification are as described for Figure 4. (B) The blot used in Figure 4D from NDT80-IN smc6-56, containing the 0 h and 7 h time points, was stripped and re-probed with the hisU probe, which only recognizes one homolog (P2) and detects the P2×P2 IS-JM (solid line) and the P1×P2 IH-JM (dashed line) as indicated in the schematic drawing. The grey square indicates the region that has been enlarged and enhanced to better visualize the JM spots in the lower panel. Species determined based on predicted size. (TIF) [file pgen.1003898.s006.tif]

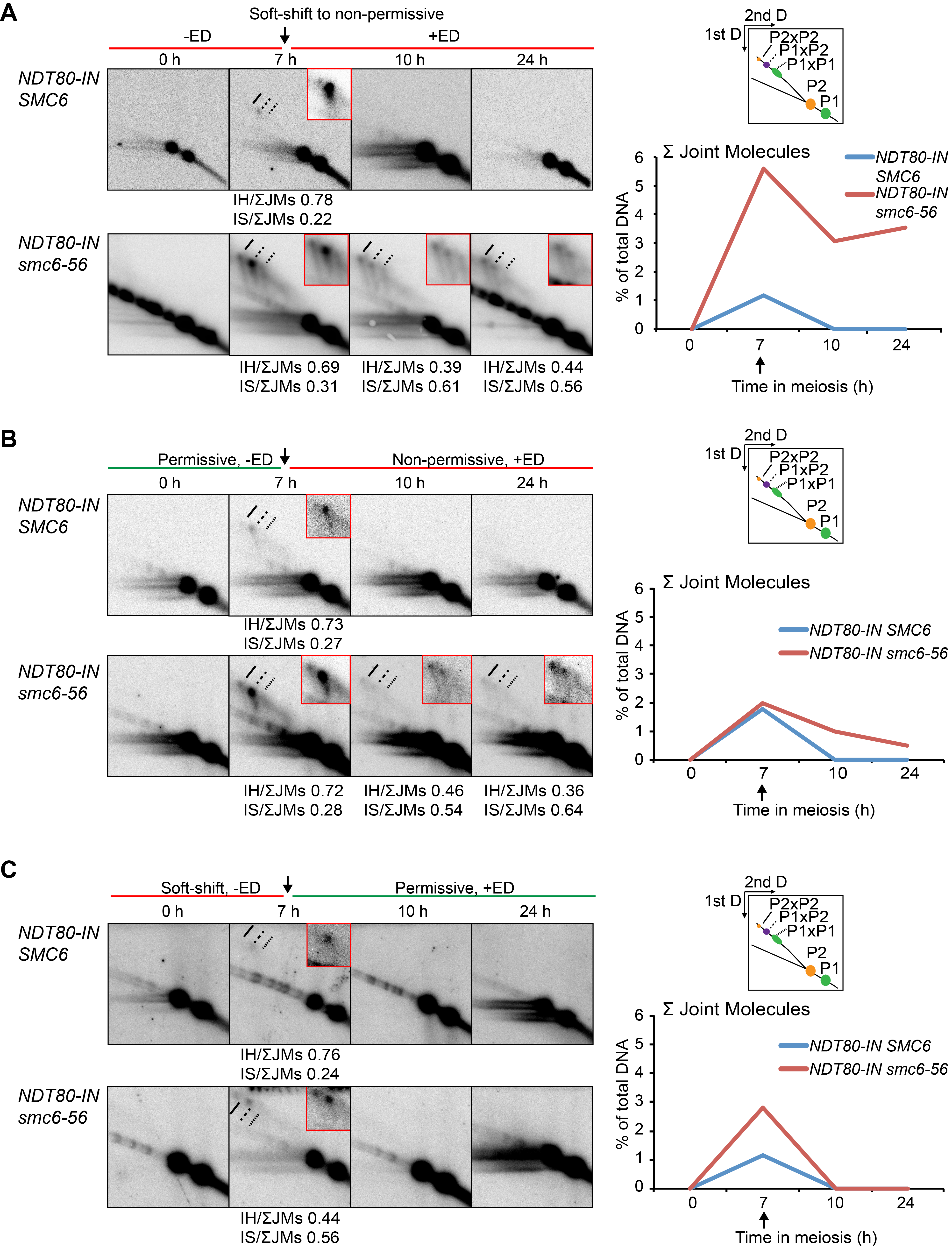

Supplement: Figure S7 — Independent experiments for two-dimensional analysis of JMs. JM analysis at indicated time points for NDT80-IN SMC6 (CB2096) and NDT80-IN smc6-56 (CB2097). Arrows above the blot images and below the graphs denote time of NDT80 induction with β-estradiol (ED). Gel conditions are as described for Figure 4 and in Materials and Methods. Ratios of IS-JM and IH-JM species given under relevant images; quantifications of shown blots given to the right of each panel. Schematic diagram represents inferred JM species. (A) JM levels and species for cells undergoing meiosis under soft-shift conditions. (B) Cells were accumulated in an ndt80 arrest at permissive temperature (green lines, −ED) until NDT80 induction when cultures were shifted to non-permissive temperature (red lines, +ED) at 7 h. (C) Cells were accumulated in an ndt80 arrest with soft-shift to non-permissive temperature (red lines, −ED). NDT80 was induced concurrently with the shift to permissive temperature (green lines, +ED) at 7 h. (TIF) [file pgen.1003898.s007.tif]

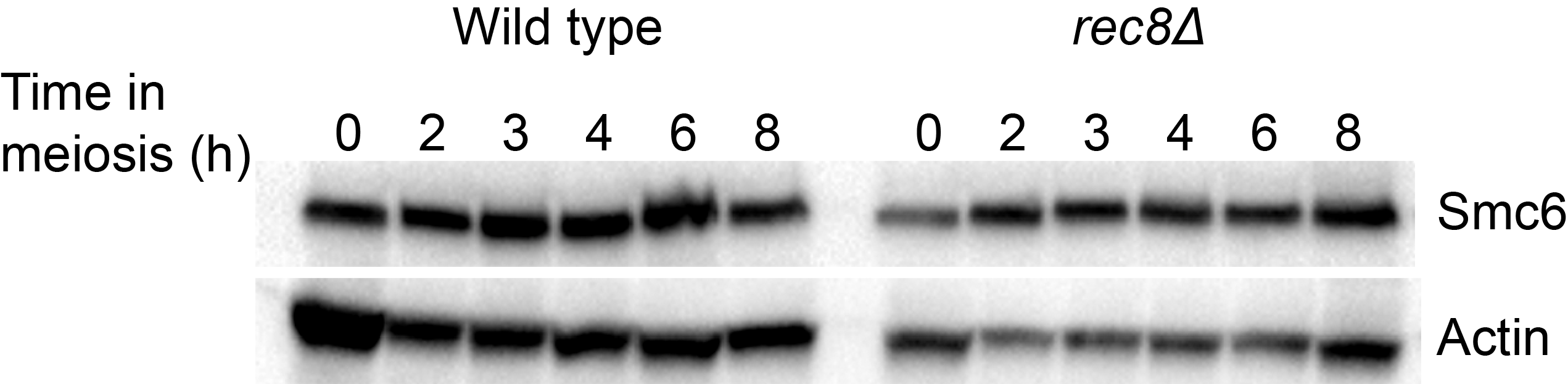

Supplement: Figure S8 — Smc6 protein levels. Western blots detecting an epitope-marked allele of SMC6 (SMC6-6HIS-3xFLAG) in a wild-type background (CB1181) and a rec8Δ background (CB1430) by extracting protein and probing for anti-FLAG as described in Text S1 at the indicated time points. Anti-actin was used as a loading control. (TIF) [file pgen.1003898.s008.tif]

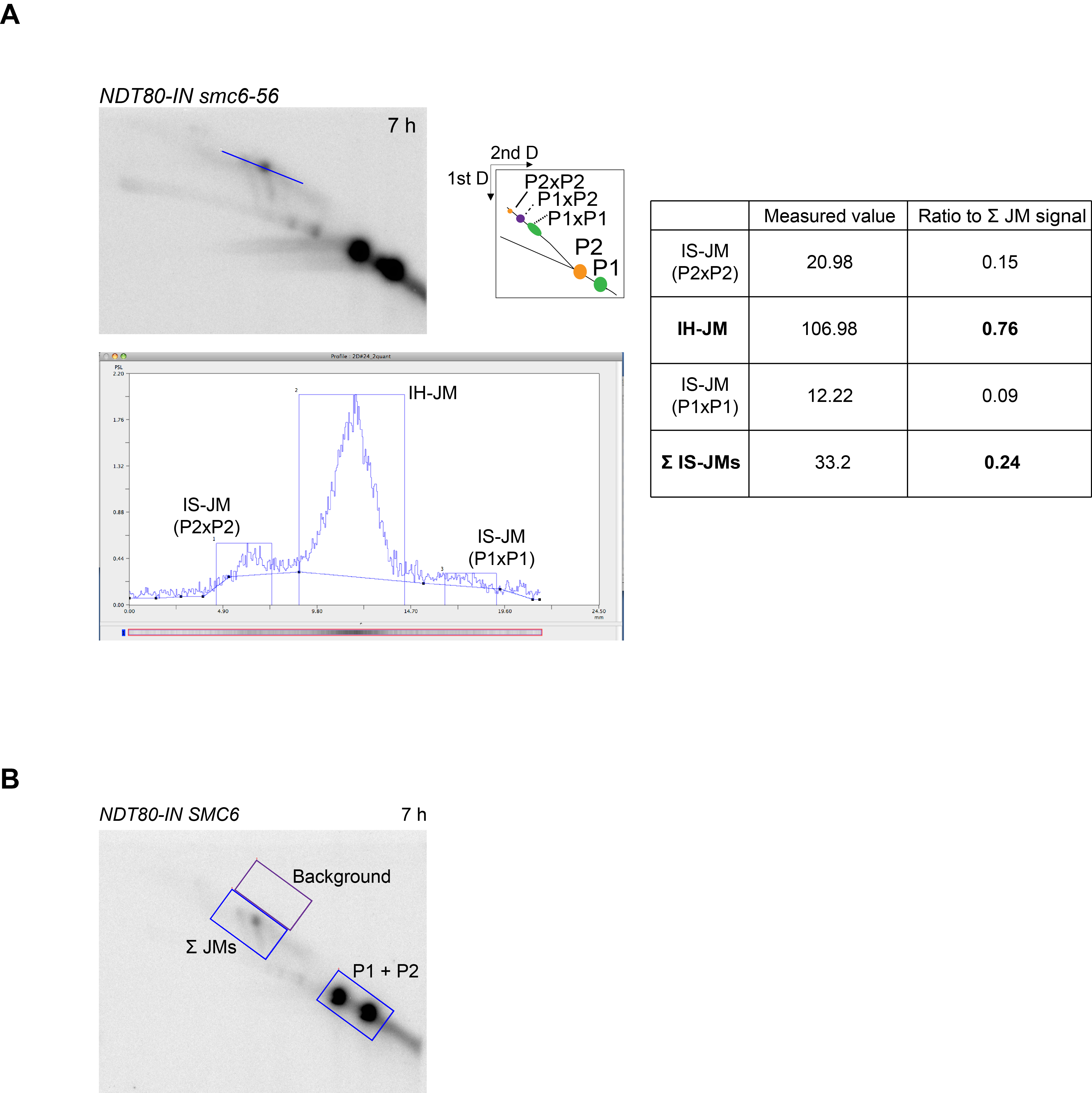

Supplement: Figure S9 — Quantification methods for calculating the ratio of JM species and total JM levels from 2D gels. (A) Determination of the ratios of IS-JMs and IH-JMs. Image is from the 7 h time point from Figure 5D, NDT80-IN smc6-56 (permissive to non-permissive). A single line was drawn to intersect the approximate centers of each JM spot using the Multi Gauge program (Fujifilm). The software generated a peak profile and the peaks representing each JM species were defined and selected after setting a threshold value as illustrated. As shown to the right, the software then generated intensity values, found from the area under each peak, and the subsequent ratios were calculated as signal/Σ JM signals. Because this method of analysis does not require using the parental bands as standards, images could be exposed for longer periods to get stronger signals in the JM region without being concerned about overexposure of the parental bands. It is also important to note that the sum of the two IS-JM signals was used for comparison. This is crucial because analyzing just one set of IS-JMs would be incorrect since the two homologs exhibit different levels of DSBs (Figure S5) [40]. Values in bold are those shown in Figure 5D. (B) Demonstration of how total JM levels were calculated from blot from the 7 h time point from Figure 6D, NDT80-IN SMC6 (non-permissive to permissive). The areas corresponding to JM and parental regions (P1+P2) were selected using equal-sized regions of interest, plus an equivalent region near the JM region corresponding to background. Percent of Σ JMs was then determined by: (measured value for JM region−measured background value)/(measured value for JM region+measured value for parental region)×100. (TIF) [file pgen.1003898.s009.tif]
