# Supplementary material for: Inhibition of the Smc5/6 Complex during Meiosis Perturbs Joint Molecule Formation and Resolution without Significantly Changing Crossover or Non-crossover Levels
Source: PLoS Genet. 2013 Nov 7;9(11):e1003898. doi: 10.1371/journal.pgen.1003898 (PMC3820751; doi:10.1371/journal.pgen.1003898)
Supplement: Table S1 — Yeast strains used in this study. All strains are derivatives of SK1 [91]. CB1017 was created by diploidizing K8379 (MATa, ho::LYS2, ura3, leu2::hisG, trp1::hisG, his3::hisG, lys2), a kind gift from the lab of Dr. Kim Nasmyth. Modifications to CB1017's genotype are indicated for each strain. Strains are MATa/α and homozygous for described loci unless otherwise indicated. The rad50S (rad50-K181 = rad50S) strains were derived from NKY1002 [79] and were a gift from Dr. Kim Nasmyth's lab. Strains used for HIS4LEU2 recombination assays were derived from NKY1303 (MATa, ho::LYS2, lys2, leu2::hisG, ura3, arg4-Bgl2, his4B::LEU2-MluII) and NKY1543 (MATalpha, ho::LYS2, lys2, leu2::hisG, ura3, his4XLEU2-MluI::BamHI-URA3, arg4-Nsp), which were originally generated in the lab of Dr. Nancy Kleckner and described in [12] and [77]. CenV-GFP and TelV-GFP strains were derived from FKY756 (MATa, ho::LYS2, promURA3::tetR::GFP-LEU2, tetOx224-URA3) and FKY4214 (MATa/alpha, ho::LYS2, lys2, trp1, promURA3-TetR-GFP::LEU2, Bmh1::tetOx224-URA3, ura3, leu2::hisG, his3::hisG), respectively. Strains containing spo11::URA3 spo13::hisG were derived from FKY1725 (MATa, ho::LYS2, lys2, spo11::URA3, spo13::hisG, trp1::hisG, leu2, his3::hisG, ura3). The strains used for JM and CO/NCO detection are descendants of MLS1827 (MATalpha, ho::LYS2, lys2, arg4del(eco47III-hpa1), leu-R, ura3, his4del(Sal1-Cla1)::URA3-del(Sma1-Eco47III)-arg4-EcPal(1691)) and MLS1076 (MATa, ho::LYS2, lys2, arg4del(eco47III-hpaI), cyh2-z, ura3, leu2-RV::URA3-(Sma1-Eco47III)-[ARG4 cloned]), which were created in the lab of Dr. Michael Lichten and originally described in [11]. Strains harbouring the NDT80-IN allele were derived from FKY4453 (MATa/alpha, ho::LYS2, lys2, ura3, leu2::hisG, trp1::hisG, his3::hisG, pGAL-NDT80::TRP1, ura3::pGPD1-GAL4(848).ER::URA3). The NKY, FKY and MLS strains are kind gifts from the lab of Dr. Franz Klein. (PDF) [file pgen.1003898.s010.pdf]

Strain	Genotype	Origin
CB1017	*ho::LYS2, lys2, ura3, leu2::hisG, his3::hisG, trp1::hisG*	K8379
**Strain**	**Additions to CB1017 genotype**	
CB46	*REC8-3HA-URA3*	This study
CB58	*rad50-K181-URA3*	NKY1002
CB319	*SMC6-13Myc-kanMX*	This study
CB1032	*smc6-56*	This study
CB1181	*SMC6-6HIS-3xFlag-kanMX*	This study
CB1183	*arg4-Bgl2/arg4-Nsp, his4B::LEU2-MluI/his4xLEU2-MluI::BamHI-URA3*	This study
CB1197	*promURA3::tetR::GFP-LEU2, tetOx224-URA3, heterozygous CenV-GFP*	This study
CB1248	*smc6-56, promURA3::tetR::GFP-LEU2, tetOx224-URA3, heterozygous CenV-GFP*	This study
CB1301	*smc6-56, spo11::hphMX*	This study
CB1302	*spo11::hphMX*	This study
CB1303	*smc6-56, arg4-Bgl2/arg4-Nsp, his4B::LEU2-MluI/his4xLEU2-MluI::BamHI-URA3*	This study
CB1346	*ScREC8-3HA-URA3, smc6-56*	This study
CB1360	*smc6-56, rad50-K181-URA3*	This study
CB1361	*REC8-3HA-URA3, SMC6-13Myc-kanMX*	This study
CB1411	*rec8::kanMX, SMC6-13Myc-kanMX*	This study
CB1426	*smc6-56, pURA3-TetR-GFP-LEU2, Bmh1::tetOx224-URA3, heterozygous TelV-GFP*	This study
CB1427	*pURA3-TetR-GFP-LEU2, Bmh1::tetOx224-URA3, heterozygous TelV-GFP*	This study
CB1430	*SMC6-6HIS-3xFlag-kanMX, rec8::URA3*	This study
CB1465	*smc6-56, spo11::URA3, spo13::hisG*	This study
CB1466	*spo11::URA3, spo13::hisG*	This study
CB1510	*kanMX-pCLB2-3HA-NSE4*	This study
CB1511	*kanMX-pCLB2-3HA-NSE4, spo11::hphMX*	This study
CB1723	*smc6-56, pGAL-NDT80-TRP1, ura3::pGPD1-GAL4(848).ER::URA3*	This study
CB1753	*pGAL-NDT80-TRP1, ura3::pGPD1-GAL4(848).ER::URA3*	This study
CB1754	*kanMX-pCLB2-3HA-SMC5, spo11::hphMX*	This study
CB1872	*kanMX-pCLB2-3HA-SMC5*	This study
CB2053	*kanMX-pCLB2-3HA-NSE2*	This study
CB2067	*kanMX-pCLB2-3HA-NSE2, spo11::hphMX*	This study
CB2059	*rad50-K181-URA3, his4xLEU2-MluI::BamHI-URA3/ his4B::LEU2-MluI, arg4-Nsp/arg4-Bgl2,*	This study
CB2060	*smc6-56, rad50-K181-URA3, his4xLEU2-MluI::BamHI-URA3/ his4B::LEU2-MluI, arg4-Nsp/arg4-Bgl2*	This study
CB2096	*arg4del(eco47III-hpa1), leu-R/ leu2-RV::URA3-(Sma1-Eco47III)-[ARG4cloned], HIS4/his4del(Sal1-Cla1)::URA3-del(Sma1-Eco47III)-arg4-EcPal(1691), cyh2-z, ura3, pGAL-NDT80-TRP1, ura3::pGPD1-GAL4(848).ER::URA3*	This study
CB2097	*smc6-56, arg4del(eco47III-hpa1), leu-R/leu2-RV::URA3-(Sma1-Eco47III)-[ARG4cloned], HIS4/his4del(Sal1-Cla1)::URA3-del(Sma1-Eco47III)-arg4-EcPal(1691), cyh2-z, ura3, pGAL-NDT80-TRP1, ura3::pGPD1-GAL4(848).ER::URA3*	This study
CB2272	*hop1::natMX, arg4del(eco47III-hpa1), leu-R/leu2-RV::URA3-(Sma1-Eco47III)-[ARG4cloned], HIS4/his4del(Sal1-Cla1)::URA3-del(Sma1-Eco47III)-arg4-EcPal(1691), cyh2-z, ura3, pGAL-NDT80-TRP1, ura3::pGPD1-GAL4(848).ER::URA3*	This study

Table S1
