# Supplementary material for: Inhibition of the Smc5/6 Complex during Meiosis Perturbs Joint Molecule Formation and Resolution without Significantly Changing Crossover or Non-crossover Levels
Source: PLoS Genet. 2013 Nov 7;9(11):e1003898. doi: 10.1371/journal.pgen.1003898 (PMC3820751; doi:10.1371/journal.pgen.1003898)
Supplement: Text S1 — Supplementary methods and references. (DOCX) [file pgen.1003898.s011.docx]

**Supplementary Methods**

**Western blotting**

Protein was prepared from meiotic samples using the TCA method as described [[1](#_ENREF_1)]. Pre-cast 7.5% Criteron TGX gels from BioRad were run and blotted according to the manufacturer’s recommendations using the BioRad system. Blots were blocked for 30 minutes in 5% milk/PBS/0.01% Tween. After splitting the membrane according to size, the blots were incubated with the appropriate primary antibody diluted in blocking buffer for 1 hour at room temperature. Membranes were washed 4 x 5 minutes in PBS/Tween and incubated with secondary antibody diluted in blocking buffer for 30 minutes at room temperature. Washing steps were repeated and signals were detected using the chemiluminescent Clarity Western ECL substrate kit with camera and ImageLab software from BioRad. Primary antibodies were as follows: the Smc6-containing portion was probed using a 1:1000 dilution of mouse anti-FLAG (Sigma) and the actin-containing blot was probed using a 1:1000 dilution of mouse anti-beta-Actin (Abcam). The secondary antibody for both blots was horseradish peroxidase-conjugated goat-anti-mouse (Life Technologies) used at 1:2000.

**Whole-chromosome DSB mapping**

Cells were collected from synchronous meiotic cultures and treated for DNA isolation in agarose plugs for chromosome-level DSB mapping as described [[2](#_ENREF_2)]. Chromosomes were separated using a pulsed-field gel electrophoresis system (BioRad) and the DNA was transferred to a nylon membrane and hybridized as described in Materials and Methods in the main text. Chromosome IV was detected using a probe specific for the *SLY1* gene at the end of the left arm of the chromosome.

**Other methods**

FACS scanning was done as described [[3](#_ENREF_3)]. To re-probe Southern blots of two-dimensional gels for homolog-specific JM detection, membranes hybridized with the argD probe were stripped by placing in 1% boiling SDS and allowed to cool to room temperature for approximately 1 hour. Stripping was confirmed after overnight exposure to imaging plates. Membranes were then pre-hybridized and hybridized with the hisU probe as described in Materials and Methods in the main text.

**Supplementary References**

1. Foiani M, Marini F, Gamba D, Lucchini G, Plevani P (1994) The B subunit of the DNA polymerase alpha-primase complex in *Saccharomyces cerevisiae* executes an essential function at the initial stage of DNA replication. Mol Cell Biol 14: 923-933.

2. Murakami H, Borde V, Nicolas A, Keeney S (2009) Gel electrophoresis assays for analyzing DNA double-strand breaks in *Saccharomyces cerevisiae* at various spatial resolutions. Methods Mol Biol 557: 117-142.

3. Michaelis C, Ciosk R, Nasmyth K (1997) Cohesins: chromosomal proteins that prevent premature separation of sister chromatids. Cell 91: 35-45.
